# Tumor-induced neurogenesis and immune evasion as targets of innovative anti-cancer therapies

**DOI:** 10.1038/s41392-020-0205-z

**Published:** 2020-06-18

**Authors:** Rodolfo Daniel Cervantes-Villagrana, Damaris Albores-García, Alberto Rafael Cervantes-Villagrana, Sara Judit García-Acevez

**Affiliations:** 1 Department of Pharmacology, Center for Research and Advanced Studies of the National Polytechnic Institute (CINVESTAV-IPN), 07360 Mexico City, Mexico; 2 Department of Environmental Health Sciences, Florida International University (FIU), Miami, Florida 33199 USA; 3 Laboratorio de investigación en Terapéutica Experimental, Unidad Académica de Ciencias Químicas, Área de Ciencias de la Salud, Universidad Autónoma de Zacatecas (UAZ), Zacatecas, México; 4 Dirección de Proyectos e Investigación, Grupo Diagnóstico Médico Proa, 06400 CDMX Cuauhtémoc, México

**Keywords:** Cancer microenvironment, Drug development, Tumour immunology, Cancer microenvironment, Drug development

## Abstract

Normal cells are hijacked by cancer cells forming together heterogeneous tumor masses immersed in aberrant communication circuits that facilitate tumor growth and dissemination. Besides the well characterized angiogenic effect of some tumor-derived factors; others, such as BDNF, recruit peripheral nerves and leukocytes. The neurogenic switch, activated by tumor-derived neurotrophins and extracellular vesicles, attracts adjacent peripheral fibers (autonomic/sensorial) and neural progenitor cells. Strikingly, tumor-associated nerve fibers can guide cancer cell dissemination. Moreover, IL-1β, CCL2, PGE_2_, among other chemotactic factors, attract natural immunosuppressive cells, including T regulatory (Tregs), myeloid-derived suppressor cells (MDSCs), and M2 macrophages, to the tumor microenvironment. These leukocytes further exacerbate the aberrant communication circuit releasing factors with neurogenic effect. Furthermore, cancer cells directly evade immune surveillance and the antitumoral actions of natural killer cells by activating immunosuppressive mechanisms elicited by heterophilic complexes, joining cancer and immune cells, formed by PD-L1/PD1 and CD80/CTLA-4 plasma membrane proteins. Altogether, nervous and immune cells, together with fibroblasts, endothelial, and bone-marrow-derived cells, promote tumor growth and enhance the metastatic properties of cancer cells. Inspired by the demonstrated, but restricted, power of anti-angiogenic and immune cell-based therapies, preclinical studies are focusing on strategies aimed to inhibit tumor-induced neurogenesis. Here we discuss the potential of anti-neurogenesis and, considering the interplay between nervous and immune systems, we also focus on anti-immunosuppression-based therapies. Small molecules, antibodies and immune cells are being considered as therapeutic agents, aimed to prevent cancer cell communication with neurons and leukocytes, targeting chemotactic and neurotransmitter signaling pathways linked to perineural invasion and metastasis.

## Introduction

Most cancers emerge from epithelial cells that suffer oncogenic mutations in the coding sequence of proteins normally controlling cell proliferation and survival.^1^ Driving genetic alterations that cause cancer occur associated to multiple external factors, including chemicals, toxins, radiation, and viral infection.^2^ Individual genetic background and conditions that affect homeostatic circuits are recognized as predisposing factors.^2^ Tumor growth and dissemination involves not only the proliferative and invasive abilities of transformed cells but also the active contribution of multiple cell lineages that turn bad under the influence of oncogenic signals.^3^ In patients, the immune and nervous systems are commonly coopted by tumors to favor cancer progression.^4–6^ At metastatic stage, the deadliest phase of cancer progression, cancer cells access the systemic circulation, move and implant in distant organs where favorable substrates allow cancer cell colonization and expansion.^7^ In the process, reciprocal communication between immune and nervous systems correlates with bad prognosis.^8,9^ The function of target organs is compromised causing systemic failure that kills most patients with metastatic cancers.^7^ Thus, understanding the cellular and molecular basis of communication among multiple cells within tumoral microenvironments emerges as the focus of basic and translational studies.

Uncontrolled cell division and altered patterns of gene expression lead cell transition into mesenchymal phenotypes.^10^ Aberrant characteristics of malignant tissues are further exacerbated by non-transformed cells that join the stroma of growing tumors in response to chemotactic signals.^5^ As they multiply in an uncontrolled manner, malignant cells form small tumor masses that require nutrients and oxygen to continue their expansion.^11^ Cancer cells at the center of millimetric tumors respond to local hypoxic conditions activating signaling pathways that promote synthesis and release of chemokines and growth factors the transform the local environment.^11^ Immune, endothelial, and neuronal, among other cell types, express receptors that respond to these oncogenic cues.^12–17^ Following chemotactic factors, they are recruited to primary tumors and metastatic niches becoming part of complex communication circuits that exacerbate the oncogenic process.^5^ Malignant cells invade surrounding tissues, either displacing normal cells or hijacking them to integrate into the stroma where their activities are redirected to benefit tumor growth. These tumor infiltrated cells that constitute the stroma include fibroblasts,^4^ endothelial cells, pericytes,^12,13^ bone marrow-derived cells (BMDC), tumor-associated monocytes and macrophages,^14–16^ endothelial progenitor cells (EPC),^18–20^ T regulators (Treg),^21^ myeloid-derived suppressor cells (MDSCs),^22^ and neuronal extensions;^17^ among other diverse components of the neuroimmune axis and many other non-related lineages. Eventually, cancer cells exhibiting invasive and anchorage-free survival properties disseminate and establish metastatic tumors.^23,24^ In the process, newly formed capillaries not only maintain the supply of oxygen and nutrients but also provide escape routes for metastatic dissemination.^7^ Strikingly, nerve fibers also serve as tracks guiding cancer cell migration.^25^

Targeting communication between tumor cells and the adjacent vasculature is the basis of anti-tumor angiogenesis therapies.^26^ Effectiveness varies depending on tumor type and resistance is an emerging problem.^26^ Various cell populations within the tumor stroma might contribute to drug resistance and increased cancer aggressiveness.^27^ Therefore, to achieve therapeutic efficacy, translational studies are focusing on the immune system which, instead of fighting transformed cells, is locally suppressed in the tumor surroundings.^28^ Immunosuppressive mechanisms displayed by cancer and stroma cells are being studied with the ultimate goal to therapeutically rescue immune cells to fight cancer. More recently, the nervous system, known to be compromised in cancer patients, is being revealed as a participant of cancer progression.^29^ Particularly, tumor-induced neurogenesis joins angiogenesis and immunosuppression as aberrant processes exacerbated within the tumor microenvironment.

### Cell communication networks in cancer

**Fig. 1:**
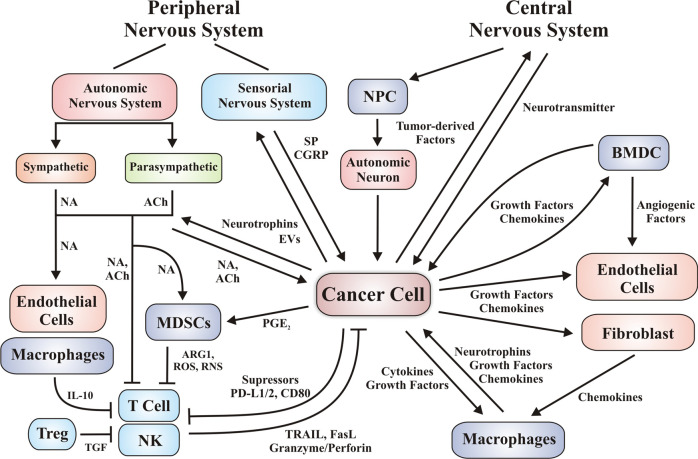
Oncogenic communication networks link tumor cells with the neuro-immune-vascular systems. Representative communication networks among tumor-associated stroma cells including fibroblasts, immune cells, vascular cells, and neuron fibers. Cell communication is either direct or mediated by cytokines, chemokines, growth factors, and fatty-acid-derived agonists. Tumor cells are positively regulated by the immune system and exhibit mechanisms to evade the antitumoral immune response. Additional communication networks, relevant for tumor vascularization, involve the contribution of fibroblasts, endothelial cells, pericytes and bone-marrow-derived cells including endothelial progenitor cells and Tie2-expressing monocyte/macrophages. Several populations of BMDC are recruited to the tumor microenvironment and niches, where they can differentiate to pro-tumor population as EPC, MDSCs, and macrophage-like cells, among others. Tumor-derived angiogenic factors promote migration and proliferation of adjacent vascular cells and BMDCs to create new vessels, growing with tumors. Central and peripheral nervous systems promote tumor growth, neurons release neurotransmitters with proliferative and migration/invasion properties on stroma and cancer cells. Peripheral nervous fibers (autonomic and sensorial) are attracted by the tumor microenvironment via axonogenesis. Tumor-derived factors recruit neural progenitor cells (NPC) to promote intratumor neurogenesis. The direction/effect arrows indicate potential targets that might be modulated by specific antagonists or agonists. Intratumor sympathetic fibers are associated in early phases of cancer triggering an angiogenic switch via adrenergic signaling. In later phases, parasympathetic fibers contribute to stimulating cancer cells to invasion and metastasis. BMDC bone-marrow-derived cell

Oncogenic communication networks established within the tumor microenvironment also exhibit systemic effects via tumor-derived mediators leaked to the circulation (Fig. 1). Invariably, some normal adjacent cells (as fibroblasts and endothelial,^4,12,13,30^ as well as distant cells (as bone marrow-derived cells^31,32^ respond as tumor subordinates, facilitating neoplastic progression. Under the influence of cancer cells, these normal (non-transformed) cells join the tumor stroma where, responding to an aberrant microenvironment, express a repertoire of genes including those coding for soluble chemotactic factors.^33^ Besides establishing reciprocal communication with cancer cells, cells within the tumor stroma secrete factors that augment the repertoire of chemoattractants that recruit additional cell populations that join primary tumors and contribute to establish metastatic niches.^17^ Cancer progression is further exacerbated under the influence of cytokines, neurotransmitters, and neuromodulators affecting different components of the neuroimmune axis.^17^ Conclusive evidence points to a pro-tumoral role of certain relevant subsets of immune cell populations. Regarding the nervous system in the context of cancer progression, recent reports support the idea that nervous cells and soluble factors inherent to their communication also contribute to cancer progression.^6^ Therefore, translational research focuses on disrupting cellular communication as a strategy to fight tumor growth and dissemination. In the next sections, we discuss how tumor cells communicate with the immune (Section “Evasion of the immune response: aberrant communication between cancer and immune cells”) and nervous system (Sections “Reciprocal communication between cancer cells and the nervous system promotes tumor progression” and “Classical central nervous system neurotransmitters (dopamine, glutamate, and GABA) impact cancer progression”). In this context, we describe potential therapeutic targets as coadjuvants of conventional therapies.

Cancer cells evade the immune system by recruiting and controlling immune cells. Cytotoxic T cells and natural killer cells (NK) follow tumor-derived chemotactic factors to be incorporated into growing tumors.^34^ Although expected to induce apoptosis of transformed cells, cytotoxic T cells, and NK cells are instead suppressed within the tumor microenvironment either by direct contact with cancer cells or under the influence of inhibitory factors.^34^ Furthermore, infiltrated Treg cells and macrophages contribute to these immunosuppressive effects on T cells and NKs.^35^ M2 macrophages also help in the promotion of tumor angiogenesis and proliferation.^36^

Communication between tumors and the nervous system is reciprocal. Cancer patients suffer neuropathic pain.^37^ The underlying neuro-oncogenic processes include pressure on fibers as tumor volume increases,^38^ secretion of stimulatory factors on peripheral fibers with depolarizing effects,^39,40^ axon demyelination,^41^ and pathological neural plasticity induced by tumor-derived factors.^42–44^ Moreover, cancer treatments, including chemotherapy (as platinum analogs, taxanes, and vinca alkaloids) and radiation, affect the nervous system causing pain. Therefore, cancer treatments commonly include painkillers, some of them extremely potent and addictive.^38,45^ Besides being a victim of cancer growth and dissemination, the nervous system is engaged by cancer cells and tumor infiltrated leukocytes to promote tumor growth and dissemination (as described in Section “Reciprocal communication between cancer cells and the nervous system promotes tumor progression”). For instance, a rat model of breast and bone cancer in which persistent pain coincided with tumor growth, served to reveal a mechanistic link between pain and tumor growth.^46^ These studies demonstrated antitumoral effects of drugs with anesthetic (bupivacaine) and analgesic (morphine) properties.^46^ These findings were interpreted as indicative of a pro-tumoral role of active peripheral fibers involved in neuropathic pain, which putatively release pro-tumoral factors. Thus, the vicious tumor-promoting circuit is initiated by cancer cells that release axonogenic neurotrophic factors, directly communicating the tumor stroma with the peripheral nervous system (PNS). As a consequence of tumor innervation, neuromediators released by tumor-associated fibers promote cancer cell proliferation and migration.^47^ In addition, sympathetic and parasympathetic fibers release noradrenaline and acetylcholine (ACh), among other neuromodulators, within the tumor and lymphoid organs to decrease anti-tumor immunological response.^48^

A variety of cell lineages within growing tumors are integrated into aberrant communication networks based on multiple chemotactic agonists secreted by cancer and stroma cells.^30,49^ Major chemoattractants include chemokine (C–C motif) ligand 2 (CCL2) and stromal-derived factor 1 (SDF-1/CXCL12) that recruit bone marrow-derived cells and M2 macrophages, upon the actions of CCR2 and CXCR4 receptors, respectively.^49^ In addition, prostaglandin E_2_ (PGE_2_) stimulates immunosuppressor MDSC cells which, as an evasion mechanism within the tumor microenvironment, arrest immune cell maturation, sustaining local immunosuppression.^22^ Thus, G protein-coupled receptors (GPCRs), the targets of these agonists, and their intracellular signaling hardware, play a prominent role in cancer. Moreover, GPCRs and their signaling transducers have been revealed as driving oncogenes themselves. Examples include activating mutations in GPCRs (e.g. CysLT_2_R-L129Q in uveal melanoma^50^) and heterotrimeric G proteins (e.g. Gαq/11-Q209L^51^ in uveal melanoma, Gαs-R201C in pancreatic cancer,^52^ and Gβ1-K57E/N/T, Gβ1-I80N/T or Gβ1-K89E/T in leukemias^53^), as well as changes in their expression and signaling properties.^12,13,54–56^ However, although targeted anti-cancer therapeutic strategies are commonly used towards tyrosine kinase-linked receptors (using kinase inhibitors and humanized antibodies together with cytotoxic/cytostatic agents), coadjutant therapy targeting GPCRs has not been fully exploited, as it would be expected given their prominent role within the tumor microenvironment.

### Emergent relevance of extracellular vesicles in oncogenic cell communication

Cell–cell communication through extracellular vesicles (EVs) as exosomes and microvesicles and its role in cancer progression has been amply discussed in previous reviews.^57,58^ Leukocyte activity in favor of tumor growth might be mediated by signaling elements incorporated by fusion of EVs. Given the diversity of proteins and other molecules transferred by this mechanism, the range of possibilities to explore therapeutic alternatives is enormous. Hypothetically, all communication networks could be modulated by EVs. For instance, tumor cells release MET^+^-exosomes that target endothelial progenitor cells. When fused to target membranes, tumor-derived exosomes enable target cells to respond to hepatocyte growth factor (HGF). In this way, tumor-derived exosomes actively contribute to tumor vascularization and growth.^31^

Targeting the exosome communication system has been postulated as a potential therapeutic strategy to fight metastasis. As cancer progression markers, exosomes with specific integrins predict metastatic organotropism.^59^ Tumor-derived exosomes contain integrins that prepare organ-specific sites where metastatic niches are established.^59^ These exosomes are uptaken by targeted resident cells like fibroblasts, macrophages, epithelial, and endothelial cells where they activate Src signaling and pro-inflammatory S100 gene expression.^59^ In murine breast cancer models, specific integrins, transferred by exosomes, determine the target organs where metastatic niches are prepared. The proposed model postulates that cancer cells release exosomes having α_6_β_4_ and α_6_β_1_ integrins that are incorporated at lungs, preparing the ground to receive metastatic cancer cells. In the case of liver metastasis, an equivalent effect has been experimentally attributed to exosomal α_v_β_5_ integrins.^59^ Experiments using exosomes collected from knockdown cancer cells, having reduced expression of targeted integrins, resulted on decreased exosome uptake at the target organ and reduced metastasis.^59^ Given their tropism for metastatic niches, engineered extracellular vesicles might serve as drug delivery systems. Microbubbles have been designed as microcapsules containing chemotherapeutic drugs, either transported inside or at the microbubble surface.^60^ Doxorubicin, paclitaxel, docetaxel, and carmustine are amongst the drugs tested. Also, ultrasound-induced destruction of microbubbles has been introduced to further control drug delivery. Drug-loaded microbubbles, sensitive to ultrasound-controlled release, can deliver drugs at primary tumors and metastatic sites. This noninvasive tool, inspired by the mechanisms of exosomal communication, will likely increase the power of targeted therapies.^61^

## Evasion of the immune response: aberrant communication between cancer and immune cells

The immune system dictates the fate of carcinogenic processes. Normally, CD8^+^ T cells^62^ and NK cells^63^ are endogenous vigilantes that destroy transformed cells using granzyme and perforin as weapons. During immunosurveillance, NK and T cells expressing FasL induce apoptosis of cancer cells having functional Fas receptors (a death receptor).^64–66^ However, cancer cells with altered Fas receptors evade the immune response.^64,67^ Besides these survival mechanisms, cancer cells can activate local immunological tolerance by overexpressing certain ligands, such as programmed cell death-ligand 1 (PD-L1), that keeps NK and T cells under their control.^68^

**Fig. 2:**
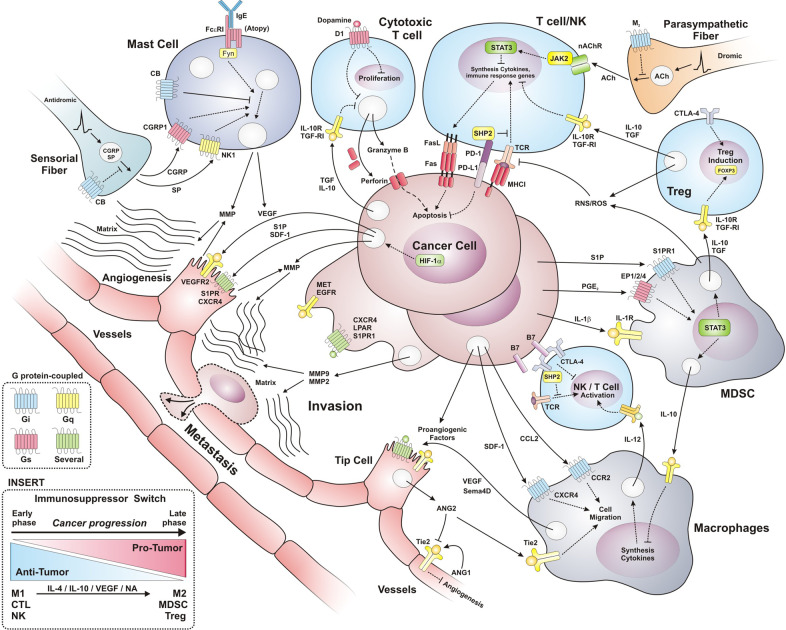
Oncogenic communication between cancer cells and tumor-associated stroma cells: immunosuppressive and proangiogenic switches. Tumor cells secrete a wide variety of factors that promote the recruitment of different cell types. The immune response evasion occurs by cell–cell interaction through transmembrane proteins as PD-L1/PD-1 and B7/CTLA-4, inhibiting cytotoxic activity. Tumor-derived factors recruit immunosuppressive cells (M2 macrophages, MDSCs, and Tregs) and promote the transition from anti-tumor to pro-tumor cells including M1 to M2 macrophages. Autonomic and sensorial fibers release neurotransmitters and neuropeptides that regulate the immune response. Parasympathetic fibers release acetylcholine, thus inhibiting immune response via nicotinic receptors, while sensorial fibers release substance P and CGRP to activate mast cells and blood vessels. To provide nutrients to the tumor, pro-angiogenic cell communication is required. Release of factors as VEGF, ANG2, CXCL12, and S1P by tumor cells, leukocytes (macrophages and mast cells), and tumor-associated fibroblasts provides an enriched microenvironment proper for tumor vascularization. The insert shows the immunosuppressor switch where in early phase of tumor development cells with anti-tumor functions are recruited, including M1 macrophages and cytotoxic T lymphocyte (CTL); yet they are progressively transformed and attract immunosuppressor and pro-tumor cells. In late phases of cancer these pro-tumor populations are enriched, correlating with high aggressiveness and low survival

Mechanistically, survival signaling in T cells is repressed by intracellular phosphatases activated by PD-1 upon interaction with PD-L1 (and also PD-L2) expressed on the surface of malignant cells.^68^ Furthermore, immunosuppressive cells are recruited by cancer cells to reinforce their anti-immune mechanisms, thus contributing to tumor growth and expansion. Cell populations with immunosuppressive effects include regulatory T lymphocytes,^69^ MDSCs^70^ and M2 macrophages,^5^ as shown in Fig. 2.

The immune response is modulated by fatty acid-derived factors, including resolvins, pronectins, lipoxins, and endocannabinoids, among others, released by cells involved in inflammation resolution mechanisms;^71^ In addition, acetylcholine, known to be involved in the cholinergic reflex, activates α7 nicotinic receptor in immune cells, triggering immunosuppressive JAK2/STAT signaling.^71^ Endocannabinoids, known as neuromodulators within the central nervous system (CNS), control secretory properties of immune cells. Therefore, they regulate the systemic availability of interleukins.^72^ Endocannabinoids such as anandamide (AEA) and 2-acylglycerol (2-AG) directly target leukocytes and also exert their neurological effects by suppressing substance P (SP) and calcitonin gene-related peptide (CGRP) of C fibers.^72^ In fact, Gi-coupled receptors expressed in the sensorial afferents potentially inhibit secretory activities of cells within the tumor microenvironment.^73^

### PD-L1/PD-1 and CD80/CTLA-4 protein complexes at immunological synapses trigger evasion mechanisms controlled by cancer cells

Protein–protein interactions established by direct contacts between cancer and immune cells create communication pathways that allow cancer cells to evade the immune response. Cancer cells hijack the regular mechanisms by which the immune system limits cytotoxic T cell activity in inflammation, autoimmune response^74,75^ and tolerance,^76^ which is normally mediated by programmed cell death protein 1 (PD-1; also known as CD279).^76^ Expression of this integral membrane protein is inducible in T cells, B cells and activated peripheral monocytes. PD-1 receptor is activated by two ligands with different expression patterns: PD-1 ligand (PD-L1; B7-H1) and PD-L2 (B7-DC).^76^ Both of them decrease interleukin 2 (IL-2) and interferon γ (IFN-γ) production, reducing T cell proliferation and cytotoxic effects.^76^ The PD-L/PD-1 system normally regulates the immune response. In the process of activating the immune response, PD-L1 is constitutively expressed in T cells, B cells, dendritic cells and macrophages, and up-regulated on stimulated T cells^77^; but also in parenchymal cells including, endothelial cells and islets of Langerhans.^78^ In contrast, PD-L2 is induced in dendritic cells, macrophages^77,79^ and active T cells.^80^ By activating intracellular phosphatases (as SHP2), this system suppresses the signaling of immune-response receptors as T-cell receptor (TCR).^71^

PD-1 cytoplasmic tail contains two phosphorylation-dependent motifs, an ITIM (Immunoreceptor Tyrosine-based Inhibition Motif) and an ITSM (Immunoreceptor Tyrosine-based Switch Motif), which are characteristic of the superfamily of inhibitory receptors that promote inflammation resolution.^71^ The suppressor effect of PD-1 was demonstrated with a chimeric protein composed by the extracellular domain of murine CD28 (co-receptor of TCR) fused to human PD-1 cytoplasmic tail. This chimeric receptor inhibited T cell proliferation and cytokine production.^81^ PD-1 ITSM motif serves as docking site for SHP-1 (Src-homology region 2 domain containing phosphatase-1) and SHP-2 phosphatases.^81^ A mutation at this site abrogates PD-1 suppressive effect.^81^ However, whether cancer cells activate this immunosuppressive signaling pathway in immune cells remains to be fully clarified. Overexpression of PD-L1 in cancer cells correlates with drug resistance and poor prognosis.^82–90^ Therefore, anti-PD-1 immunotherapy has gained enormous clinical relevance and its suitability in different conditions is the focus of hundreds of clinical trials (https://clinicaltrials.gov/).

Immunotherapy with anti-PD-1 antibodies increases the infiltration of CD8^+^ cytotoxic T cells into soft tissue sarcomas,^91^ melanoma,^87^ and murine renal cancer.^92^ Conventional chemotherapy and therapeutic protein kinase inhibitors are expected to improve their efficacy when combined with anti-PD-1 antibodies.^93^ Encouraging results have been reported in preclinical studies of ovarian cancer,^94^ and T-cell non-Hodgkin lymphoma.^95^ Also, anti-PD-1 monoclonal immunotherapy enhances the effect of a vaccine against hepatocellular carcinoma (GPC3-derived peptide vaccine, phase II) in patients.^96^ In certain conditions, anti-PD-1 antibodies have demonstrated to be effective even in cases of drug resistance to cytotoxic chemotherapy. For instance, pembrolizumab, a monoclonal anti-PD-1 antibody, showed antitumor effect in a patient with a solitary fibrous tumor of pleura resistant to chemotherapy. The therapeutic antibody was well tolerated and did not generate significant adverse effects over the therapeutic cycle.^97^ Overall, the proof of concept regarding the therapeutic use of anti-PD-1 monoclonal antibody is well established. It is expected to be effective in cancers where evasion of the immune system plays a fundamental role in tumor progression.

A second immunomodulatory system hijacked by cancer cells is the one composed by CTLA-4 (Cytotoxic T-Lymphocyte Antigen 4) receptor, known to be exclusively inducible expressed in T lymphocytes, and constitutively expressed in regulatory T cells (Tregs).^98^ Normally, this system attenuates effector T cells (CD4^+^CD25^-^) and enhances regulatory T cells (CD4^+^CD25^+^).^99–101^ It is physiologically activated by antigen-presenting cells expressing CD80 (also known as B7.1) and CD86 (also known as B7.2), known CTLA-4-ligands. Immunosuppressive activity of certain cancer cells is gained by expression of CD80 and CD86.^102,103^ CTLA-4 and CD28 (co-receptor of TCR) recognize the same ligands. CTLA-4 is induced after TCR activation and competes with CD28 for ligands to inhibit TCR via phosphatases as SHP-2 and PP2A.^104–106^ The interaction of CTLA-4 with phosphatase SHP2 requires a tyrosine-phosphorylated Tyr-Val-Lys-Met (YVKM) motif in the cytoplasmic tail of CTLA-4 to regulate the TCR activity; in fact, T cells, in the absence of CTLA-4 have an hyperactive TCR signaling (Fyn, Lck, ZAP-70) leading to pro-inflammatory functions.^107^ While the catalytic subunit of the serine/threonine phosphatase PP2A also interacts with the YVKM motif of CTLA-4.^105^ The phosphatase PP2A mediates CTLA-4 signaling to inhibit the activation of T cells;^108^ in fact, PP2A is a target for immunotherapy, and the inhibition of the phosphatase activity increases the cytotoxicity of intratumor lymphocytes.^109^ For PD1 and CTLA-4 receptor, it is necessary to have in vivo evidence showing how the immunosuppressive effects in several tumor microenvironments are mediated by the direct activation of phosphatases.

Partial blockade of CTLA-4 shows therapeutic potential as it increases the antineoplastic effect of non-selective cytotoxic substances contributing to tumor regression in experimental cancer models, whereas non-immunogenic tumors are resistant. However, excessive blockade of CTLA-4 with therapeutic purposes has been controversial as it can cause an autoimmune disorder due to a lymphoproliferative effect.^110^ In clinical settings, anti-CTLA-4 monoclonal antibodies (ipilimumab and tremelimumab) are particularly effective in patients with melanoma.^111,112^ Also, promising results have been obtained in the treatment of patients with refractory head and neck squamous cell carcinoma,^113^ metastatic sarcoma,^114^ metastatic colorectal cancer,^115^ small-cell lung cancer,^116^ non-small-cells lung cancer,^117,118^ metastatic renal cell carcinoma,^119^ and malignant mesothelioma.^120–122^ As combined therapy, anti-CTLA-4 improves the antitumoral effect of conventional cytotoxic substances. As mentioned before, blocking the CTLA-4 receptor increases CD4^+^ T cells activities therefore stimulating effector cells.

### Regulatory T cells (Tregs) promote the tumor growth via inhibitory cytokines

The antitumor effect of anti-CTLA-4 monoclonal antibodies is based on their ability to deplete CD4^+^/FOXP3^+^ T regulatory cell population.^123^ T regulatory cells need CTLA-4 for suppressive function, in fact, CTLA-4-deficient Tregs increases immunity against tumors in mice.^100^ Similarly, anti-PD-1 monoclonal antibodies interfere with the ability of these cells to communicate with antigen-presenting cells.^124^ Regularly, Tregs maintain immune tolerance. They are an immunosuppressive population of CD4^+^/CD25^+^ T cells, identified in 1995 by Sakaguchi et al.^35^ Further characterization of this cell population led to the identification of FOXP3, as a marker of regulatory T cells.^125,126^ FOXP3 directly suppresses IL-2 gene expression and increases CTLA-4 and CD25 expression.^127^ In addition, via secretion of inhibitory cytokines as IL-10, IL-35 (interleukin-10/-35), and TGFβ (Transforming Growth Factor-β), T regulatory cells inhibit granzyme and perforin expression in antigen-presenting cells and degrade ATP, causing energy deficiency.^128^ Several preclinical studies have reported that Treg cells play a fundamental role in tumor immunity, since depletion of this T cell population, using monoclonal antibodies against CD25^+^, prevents tumor growth.^129^ In clinical studies, increased presence of Treg cells is indicative of bad prognosis in ovarian cancer.^130^ However, in the case of colorectal cancer, controversial findings have been reported regarding whether increase of Tregs infiltrated into tumors improves or worsens the prognosis.^131^ These data suggest that further sub-classification of Tregs is required to explain differences in the outcome of various types of cancers. Additional markers might increase the effectiveness of precision immunotherapies.

Given the success of anti-CD25 antibodies preventing tumor growth in preclinical cancer models, current clinical trials are addressing the blockade of Treg CD25^+^ receptor with daclizumab, a humanized monoclonal antibody. This antibody was tested in patients with metastatic melanoma together with vaccination of dendritic cells. As a result, Treg cells in peripheral blood were depleted, but antitumor effector T response was not achieved.^132^ In contrast, daclizumab followed by vaccination potentiated the antitumor response in breast cancer patients.^133^ Since Treg cells produce TGFβ, a cytokine whose signaling promotes cancer progression and metastasis of several types of cancers, additional therapeutic efforts are oriented to target TGFβ receptors. TGFβ-dependent effects are linked to tumor-induced angiogenesis and direct immunosuppressive effects mediated by a decrease on the innate and adaptive antitumor immune response.^134^ Preclinical studies on the therapeutic potential of galunisertib (LY2157299), an inhibitor of TGFβ serine/threonine kinase type 1 receptor (TGFβ-RI), have shown anti-tumor effects in neuroblastoma and hepatocellular carcinoma, showing an increase on natural killer cells;^135^ and modulating the expression of CD44^+^,^136^ respectively. Clinical trials are underway to explore the use of this kinase inhibitor in recurrent glioblastoma,^137^ and advanced pancreatic cancer,^138^ among others.

### MDSCs and macrophages contribute to evade anti-tumor responses

Immunosuppressive cell populations contribute to antitumor evasion. Myeloid-derived suppressor cells (MDSCs) were first described in 1987 in a mouse lung cancer model. In lung tumors, they were recognized as frequent infiltrating immature myeloid cells and their immunosuppressive functions were postulated. However, these initial, visionary experiments, were not further pursued.^139^ It is now established that MDSCs are myeloid cells similar in their origin to macrophages, granulocytes, and dendritic cells.^140^ This heterogeneous cell population emerge under pathological conditions such as cancer, inflammatory diseases, autoimmune diseases and chronic viral infections, conditions that interrupt the maturation process normally occurring in this cell population.^141,142^ MDSCs, identified in human spleen, are classified in two main subpopulations: granulocytic- and monocytic-MDSCs. These cells express several plasma membrane markers (Lin^−^, CD11b^+^, CD33^+^, HLA-DR^−^); additionally, CD14^+^, CD15^+^ characterizes granulocytic MDSCs, whereas monocytic MDSCs are CD14^+^, CD15^−^.^143^ Immunosuppressive effects of MDSC are mediated by three major mechanisms: (1) Reactive oxygen species (ROS) that block macrophages and dendritic cell differentiation;^144,145^ (2) inducible nitric oxide synthase (iNOS) associated with decreased T cell expansion and proliferation capabilities;^146^ and (3) Arginase-1 (Arg1) that diminish T-cell metabolism and promotes TCR nitrosylation, ultimately leading to apoptosis.^147^ Other mechanisms of immune regulation by MDSCs have been described, they include alterations in antigen presentation, T cell signaling, immunosuppressive and pro-apoptotic factor production, induction of inhibitory signaling cascades and recruitment of regulatory T cells.^148^ In response to tumor antigens presented as peptides on the surface of MDSCs, they inhibit IFN-γ production by CD8^+^ T cells.^149^ In vivo, MDSCs induce antigen specific tolerance in T lymphocytes.^150^ Clinical studies revealed that increased levels of MDSC correlate with poor prognosis in cancer patients.^151^ After exacerbated responses, these cells contribute to restore homeostasis.

Therapeutic reduction of MDSCs population would diminish immunological antitumor tolerance. Conventional chemotherapy contributes to this goal. For instance, in gastric cancer models, cytotoxic chemotherapy with ipirubicin and paclitaxel decrease MDSCs population as a consequence of anti-proliferative and pro-apoptotic effects in which the MAPK and NFκB signaling pathways are involved.^152^ Also, acute lymphocytic leukemia patients treated with chemotherapeutic molecules have less suppressor cells, which contributes to a better prognosis.^153^ Breast cancer patients expressing IL-17, and a STAT3 activated pathway, have less tumor-infiltrated MDSCs,^154^ raising possibilities to target IL-17 as a therapeutic alternative. Altogether, these studies highlight the importance of studying immunosuppressive cell populations as targets of therapeutic alternatives against cancer. Prostaglandin E_2_ (PGE_2_) induces the differentiation of MDSC cells via E-prostanoid (EP) receptors. Differentiation is blocked by antagonists of prostaglandin receptors: EP4 (AH23848), EP1/EP2 (AH6809); and cyclooxygenase-2 inhibitor (COX2 inhibitor SC58236). In a preclinical tumor model using 4T1 mammary carcinoma cells, EP2-deficient mice showed decreased tumor growth and MDSC infiltration; similarly, wild-type mice treated with COX2 inhibitor showed reduced primary tumor growth and delayed MDSC accumulation.^22^ In cancer therapy, EP receptor antagonists and COX2 inhibitors may attenuate the accumulation of MDSCs and their contribution in tumor growth.

Tumor-associated macrophages (TAMs), as Tregs and MDSCs, are infiltrated within the microenvironment of most solid tumors.^155^ TAMs express PD-L1 which, as previously described, can directly decrease T cell activation. Normally, macrophages produce matrix metalloproteinases (MMPs) involved in physiological angiogenesis and tissue repair.^156^ In the case of tumor-associated macrophages, those with M1 phenotype exhibit a tumor suppressor role,^157^ whereas M2 macrophages have immunosuppressive effects propitiating tumor growth and metastasis. Within the tumor microenvironment, acquisition of M2 phenotype is promoted in response to IL-10 and IL-4 cytokines, in addition to some growth factors such as vascular endothelial growth factor A (VEGF-A)^158,159^ and catecholamines (noradrenaline and adrenaline) released by tumor-associated sympathetic fibers and adrenal glands.^160^ As with other immunosuppressive cell populations, reducing M2 macrophages likely improves patient prognosis, as it has been recently shown in skin cancer patients. Clinical trials testing emactuzumab, which targets colony-stimulating factor receptor 1 (CSF-1R) decreasing the M2 macrophage population, have reportedly improved prognosis of skin cancers patients, like those with melanoma.^161,162^

Macrophages are heterogeneous and can be functionally polarized in pro-inflammatory M1 macrophages (classical activation by IFNs, Toll-like receptor) or anti-inflammatory M2 macrophages (alternative activation by IL-4/IL-13). M1 macrophages are anti-tumor, while M2 macrophages are pro-angiogenic and immune suppressors.^163–165^ M1 and M2 differ in the expression of receptors, cytokine and chemokine production and effector function.^166^ Patients with high infiltration of M1 macrophages had better survival versus low infiltration; in contrast, high infiltration of M2 macrophages had worse overall survival versus low infiltration.^165^

Anti-tumor M1 macrophages are recruited in early phases to tumor development, but are progressively differentiated to M2 with pro-tumor effect.^167,168^ Reduced hypoxia in early phases of tumor progression allows the accumulation of M1 macrophages, increasing antigen presentation and promoting antitumor cytotoxicity by T cells. As tumor hypoxia increases, cytokine production for the anti-tumor response becomes deficient and results in tumoricidal decline and progressively macrophages acquire pro-tumor M2 functions driven by the tumor microenvironment^167,169^ including IL-4 and IL-10 ^159^. The overexpression of the p50 subunit of NFκB in macrophages promotes the re-polarization of M1 to M2, gradually atrophying the efficient anti-tumor response and switching to pro-tumor functions by accumulation of p50 homodimers. In p50 deficient mice or with restricted deficiency in bone marrow cell, it retards the growth of melanoma (B16) and fibrosarcoma (MN/MCA1) tumors.^168^ The evidence suggests that there is an immune-suppressor switch that promotes the change of populations of anti-tumor immune cells, such as M1, to populations of immunosuppressive cells such as M2 macrophages.^167^ The polarization of macrophages could be a therapeutic target, establishing immunotherapies for the accumulation of M1 macrophages with tumoricidal functions by recruiting and preventing their desensitization and switching to M2. It is necessary to clarify the ways of differentiation to M2 for a more rational therapy.

Tumors recruit cells of the immune system. Tumor-associated monocytes/macrophages are recruited into the tumor microenvironment by chemokines such as SDF-1 and CCL2. In these settings, activation of CXCR4 and CCR2 receptors promote Gi-dependent cell migration.^49^ Interestingly, the intratumor differentiation of monocyte to macrophage requires CCR2 downregulation to retain the cell in the tumor.^166^ In cancer patients, populations of Tie2-expressing monocytes/macrophages (TEMs) colonize tumors. These cells migrate in response to angiopoietin-2 (Ang2), a Tie2 ligand, released from activated endothelial cells during angiogenesis.^15^ Reciprocal communication between tumor cells and TEMs contributes to tumor progression. For instance, Tie2^+^/CD11b^+^/CD45^+^ bone-marrow-derived cells promote tumor growth in lung carcinoma mice models. BMDCs secrete factors that stimulate cancer cell migration via Gi-coupled receptors signaling via Gi→Gβγ. Chemotactic GPCRs and their Gβγ-dependent signaling effectors are essential to promote cell migration within the tumor microenvironment and at a systemic level to recruit normal somatic cells to growing tumors.^16,170^

Semaphorins are a family of membrane-associated or secreted glycoproteins, initially involved in axonal guidance, and relevant to cancer progression by modulating cell migration of leukocytes, neurons, and endothelial cells.^171^ In tumor-associated macrophages, semaphorin 4D (Sema4D), induces tumor angiogenesis and vessel maturation by binding to the plexin B1 receptor on endothelial cells, and the effect is blocked by plexin B1 antibodies, and by the c-Met inhibitor (PHA-665752). In fact, knocking out of Sema4D prevents tumor growth and metastasis in a breast cancer murine model (TSA cells).^172^

Catecholamines activate the immunosuppressor switch in the tumor microenvironment (TME) of lung cancer accumulating M2-polarized macrophages and MDSCs while decreasing antitumoral dendritic cells (DC).^160^ Tumor-associated M2 macrophages synthesize and release VEGF, promoting angiogenesis, which is induced by adrenergic signaling in macrophages.^160^ Moreover, re-polarization of M1 to M2 macrophages by tumor catecholamines leads to the synthesis and release of IL-10, an immunosuppressive cytokine for TME.^160^ The inhibition of adrenergic signaling increases the antitumor immune response via the impact on multiple leukocytes.

### Neuronal regulation by tumor-associated leukocytes

The role of the nervous system in regulating the immune response in infection and inflammatory processes is known.^173^ In the opposite direction, it has been described that mediators released from leukocyte populations can modulate the activity and prolongation of the adjacent nerve fibers and infiltrate to the tumor.^174,175^ Inflammatory mediators lead to the activation of peripheral sensory fibers that, in addition to promoting pain, lead to the release of substance P, a neuropeptide that promotes tumor growth.^46,176^ Vasodilation of peri- and intratumor vessels, as well as increased vascular permeability by tumor-derived vasoactive factors contribute to the extravasation of leukocytes to access the tumor and the intravasation of metastatic cells.^177^

Inflammation processes induce the accumulation of leukocytes and the release of pain-associated mediators, promoting neuronal plasticity and peripheral innervation.^178^ Pain research, including cancer pain, highlight the contribution of macrophages-derived neurotrophins and other non-neuronal cells, in the stimulation of nociceptors and damaged nerves, enhancing pain and generating aberrant neuromas that spontaneously depolarize, contributing to neuropathic pain.^178,179^ M2 macrophages are functionally recruited and aid in nerve repair and possibly tumor innervation, in contrast M1 functions to destroy the injured nerve.^180^ Macrophages regenerate the injured nerve through secretion of VEGF that guides the growth of new blood vessels, used by Schwann cells to migrate and guide the nerve growth,^174^ a mechanism that can be used in tumor angiogenesis and axonogenesis. In addition, Sema4D can induce neurite outgrowth,^181,171^ and this semaphorin is mainly expressed in tumor-associated macrophages promoting tumor angiogenesis,^172^ but possibly also tumor innervation.

Neurotrophins, as nerve growth factor (NGF), when released by macrophages, mast cells, and other leukocytes could be promoting to axonogenic switch for tumor innervation, as occurs in inflammatory pain^178^ and neurogenesis by recruiting brain-derived neural precursors for cancer progression (Section “Tumor neurogenesis: CNS-derived neural progenitor cells infiltrated in tumors”). Macrophages activation leads to high sensory and sympathetic innervation joint to angiogenesis in an arthritic inflammation murine model.^182^ Macrophages, neutrophils, T-lymphocytes, and mast cells express NGF.^183,184^ In damaged nerves, macrophage-derived IL-1β induces the synthesis of NGF in non-neuronal cells.^175^ There are no studies showing that neurotrophins released by tumor-associated leukocytes contribute to tumor innervation and neurogenesis in cancer, but it is hypothetically possible.^178,180^

## Reciprocal communication between cancer cells and the nervous system promotes tumor progression

**Fig. 3:**
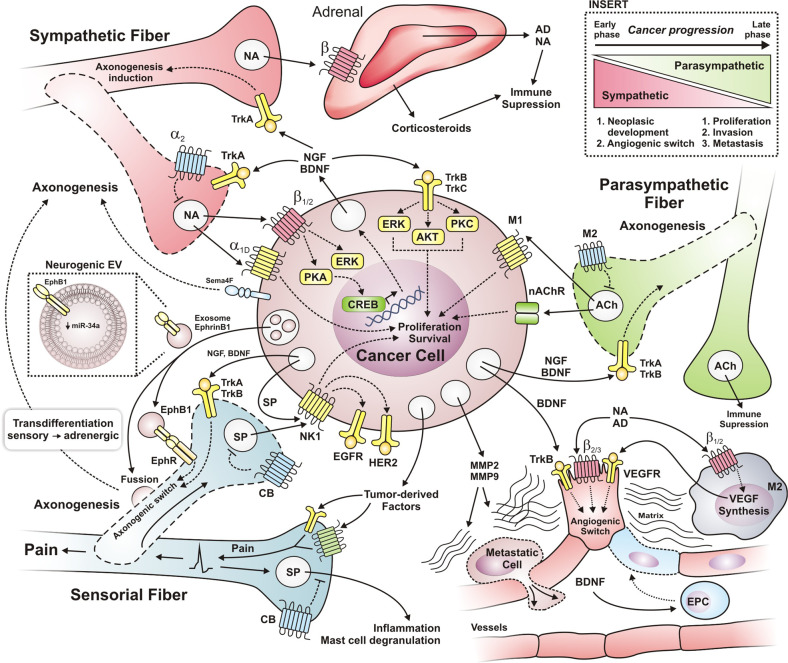
Axonogenesis is induced by oncogenic communication between cancer cells and adjacent sensorial/autonomic fibers. Tumor-derived neurotrophins (as NGF and BDNF) promote the axonogenic switch of sensorial afferent and autonomic efferent fibers derived of tumor-adjacent nerves. Then, nerve fibers innervating the tumors release factors allowing survival, proliferation, and migration of cancer cells. The autonomic fibers innervating the tumor release noradrenaline and acetylcholine, providing a direct stimulus to receptors expressed in cancer cells. Tumor-derived factors stimulate sensory fibers triggering pain, and the antidromic signals promote neuropeptides release (as SP) into the tumor, activating NK1 in cancer cells and leading to growth factor receptor transactivation via Src (EGFR, HER2). The insert shows the contribution of sympathetic and parasympathetic fibers during cancer progression. Sympathetic neurons contribute highly in early phases, the sympathetic fiber-derived noradrenaline activates an angiogenic switch in endothelial cells, promoting neoplastic development. As the contribution of sympathetic signaling decreases, there is a robust contribution of parasympathetic fibers in late phases inducing proliferation, invasion, and metastasis

Nerves promote tumor growth, invasion, and metastasis; tumor-associated-fibers are considered components of the tumor stroma.^47^ Neuropeptides or neurotrophic factors released by tumor cells promote axonogenesis to innervate the growing tumor^185^ (Fig. 3). In reciprocity, fiber-derived neurotransmitters as glutamate, GABA (γ-aminobutyric acid), noradrenaline or acetylcholine stimulate tumor cell survival, proliferation, and migration. In addition, neurotransmitters modulate pro- and anti-immune responses, also affecting the tumor microenvironment by such indirect mechanisms. Therefore, potential therapeutic alternatives might be based on stopping aberrant tumor neurogenesis and disrupting communication between cancer cells and neurons. Likely, combined with conventional anti-tumor therapies, targeting neuromediator receptors could be the basis of novel anti-neoplastic treatments in cases where tumor-induced neurogenesis is proven to be relevant for cancer progression.^17^

### Peripheral nervous system in tumor axonogenesis and perineural invasion

Tumor-derived neurotrophins as NGF and extracellular vesicles can induce tumor innervation by stimulating branching of adjacent nerve terminals, either of the somatosensory, motor, or autonomic system,^17,186^ also contributing to cancer-associated neuropathic pain.^187^ In pancreatic tumor xenografts (MIA PaCa-2 cells) chemical denervation (botulinum toxin) decreases the tumor growth and increases apoptosis.^188^ In prostate cancer, nerve density increases and correlates with cancer cell proliferation and an increase in the expression of proteins involved in survival as NFκB, c-Myc, GSK-2, PIM-2, SKP, SRF, PTEN, androgen receptor, and estrogen receptor α.^189^ Invariably, patients with densely innervated tumors will develop increased metastasis, have a poor prognosis, and decreased survival.^186,190^

Cancer cells proliferate around peripheral nerves and eventually invade them. This process, called perineural invasion (PNI), is a pathological feature of several types of cancer that correlates with reduced survival of patients.^25,191^ This process could be a druggable target as tumor-angiogenesis. Tumor cells migrate and expand along nerves (Fig. 3), as an alternative route to metastasize. In vitro, prostate tumor cells (PC3 cells) migrate along neurites branched from the dorsal root ganglia (DRG). Adrenergic (β-blockers propranolol and penbutolol) and muscarinic antagonists (atropine and hyoscine) effectively inhibit prostate cancer cell migration along neurites, suggesting that these nervous extensions provide guidance and biophysical support to facilitate cancer cell dissemination, preventing this process could improve cancer therapeutics. Sympathetic fiber-derived noradrenaline activates the β2-adrenergic receptor and induces PNI via PKA/STAT3 activation. STAT3 leads to the expression of NGF, MMP2, and MMP9 in pancreatic cancer cells so that they can migrate and invade.^192^

Pancreatic ductal adenocarcinoma (PDAC) is exacerbated by neuropsychological stress via β2-adrenergic signaling (PKA and ERK pathways). In this case, tumor cells secrete NGF and BDNF (brain-derived neurotrophic factor) stimulating nerve growth via their Trk receptors (Tropomyosin-related kinase receptors) (Fig. 3). Therefore, β2-adrenergic antagonists (ICI-118, 551, propranolol, but not atenolol) and inhibitors of Trk receptors (pan-Trk inhibitor PLX-7486), potentiate the therapeutic effect of gemcitabine, prolonging mice survival, and non-selective β-blocker treatment prolong survival of patients with PDAC.^185^ Interestingly, hyperglycemia increases cancer cell proliferation and induces NGF overexpression, promoting PNI in pancreatic cancer. Furthermore, hyperglycemia-dependent demyelination and axonal degeneration propitiate PNI.^193^ Finally, PNI induces neuropathic pain during pancreatic cancer when tumor-derived factors activate sensorial fibers to trigger pain; some of the factors that increase in cancer cells are NGF, BDNF, artemin, and glial cell-derived neurotrophic factor (GDNF), while their receptors increase in nerves.^187^

**Fig. 4:**
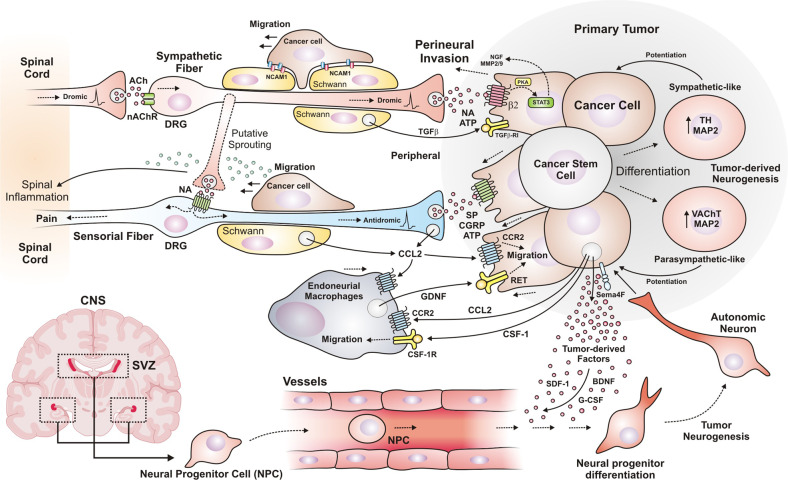
Tumor neurogenesis and perineural invasion, close and distant communication between cancer cells and neurons. In perineural invasion, cancer cells migrate in response to different mediators released by autonomic and sensory fibers. Also, tumor cells secrete CCL2 and CSF-1 to accumulate endoneurial macrophages and, at the same time, release factors that stimulate perineural invasion. Cancer stem cells have the faculty to differentiate and acquire an autonomic neuron-like phenotype generating tumor-derived neurogenesis. Also, neurons and Schwann cells release GRFα1 and GDNF (secreted by the endoneurial macrophages), activating RET in tumor cells. Besides, Schwann cells release TGFβ, increasing the aggressiveness of cancer cells through TGFβ-RI. Schwann cells drive perineural invasion, cancer cells interact directly with Schwann cells via NCAM1 to invade and migrate along nerves. Tumor-derived neurogenesis occurs when cancer stem cells differentiate to neuron-like cells, particularly to autonomic neurons that release neurotransmitters to enrich the tumor microenvironment. Tumor-induced neurogenesis is characterized by the recruitment of neural progenitor cells (NPC)-derived from the central nervous system (CNS), particularly from the subventricular zone (SVZ). NPCs travel through the bloodstream attracted by tumor-derived factors, once they infiltrate and colonize the tumor, they differentiate into functional autonomic neurons that stimulate tumor growth. DRG dorsal root ganglion, SVZ subventricular zone, CNS central nervous system, NCAM1 neural cell adhesion molecule 1, ACh acetylcholine, NA noradrenaline, SDF-1 stromal derived factor, TH tyrosine hydroxylase, VAChT vesicular acetylcholine transporter, BDNF brain-derived neurotrophic factor, CCL2 chemokine (C–C motif) ligand 2, CSC cancer stem cell, NPC neural progenitor cell

Tumor-associated nerves promote PNI because of CCL2 chemotactic actions. This chemokine is one of the most prominent factors in the tumor-associated nerve secretome. CCL2 induces cancer cell migration and PNI via CCR2 signaling.^194^ Moreover, macrophages infiltrated into pancreatic adenocarcinomas contribute to PNI. Nerve resident macrophages accumulate in the nerves invaded by tumor cells by following the gradient of CCL2 and CSF-1 recognized by CCR2 and CSF-1R receptors, respectively; CSF-1 receptor blocker (GW2580) prevents the migration of endoneurial macrophages induced by tumor-derived factors.^195^ Then, tumor cell migration is triggered by RET receptors activated by GDNF released by activated macrophages (Fig. 4). Cancer cell migration induced by macrophage-derived GDNF depends on GFRα1 co-receptor and RET, as demonstrated by the inhibitory effect observed in knock down experiments with cells lacking GFRα1 co-receptor and the use of RET inhibitor (pyrazolopyrimidine-1, PYP1). The signaling pathways controlling pancreatic cancer cell migration involve MEK1 and AKT, as indicated by the inhibitory effect of small molecules targeting these kinases.^195^ GFRα1 is a RET co-receptor that potentiates cancer cell migration and enhances PNI. Interestingly, cancer cells that lack GFRα1 still invade nerves because soluble GFRα1 and GDNF are released by neurons and their associated Schwann cells,^196^ strongly activating RET in cancer cells.^197^

Schwann cells drive PNI; cancer cells associate and use Schwann cells to invade and migrate along nerves in pancreatic and thyroid cancer (Fig. 4). In tumor nerves with PNI there is an increase in Schwann cells (GFAP^+^, Glial fibrillary acidic protein) and they intercalate with cancer cells by direct contact through NCAM1 (neural cell adhesion molecule 1). In NCAM1-deficient mice there is a decrease in Schwann-cancer cell contact and the invasion distance of nerves.^198,199^ Additionally, Schwann cells are a source of TGFβ that activate SMAD signaling in pancreatic cancer cells inducing migration, aggressiveness, and PNI, this effect is sensitive to pharmacological inhibition of TGFβ-RI (SB-431542 inhibitor).^200^

### Neurotrophins directly stimulate cancer cells and induce tumor vascularization

Neurotrophins can directly induce tumor-axonogenesis (previous section), cancer cells stimulation and tumor-angiogenesis; connecting the three processes that promote tumor growth. In oral squamous cell carcinoma (OSCC), Trk receptors are overexpressed: TrkA (for NGF), TrkB (for BDNF), and TrkC (for neurotrophin 3, NT3) receptors. Cultures of highly metastatic cells (KON and HSC-3 cells) and patient samples exhibited higher expression of TrkB and TrkC. The presence of these receptors in OSCC patients correlated with low disease-free survival,^201^ and in patients with ovarian cancer high expression of TrkB correlated with low overall and disease-free survival.^202^ However, in neuroblastoma patients, high expression of TrkA or TrkC correlates with a better prognosis.^203,204^

In tumors, paracrine and autocrine neurotrophins directly activate their receptors in cancer cells, activating PI3K/AKT, Ras/ERK, and PLCγ/PKC signaling pathways for survival and proliferation (Fig. 3).^204,205^ Ovarian cancer cells (OVCAR-3, SKOV-3, OVCA420, OVCA429, and OVCA433) overexpress TrkB, in fact, HGF induces TrkB expression. BDNF/TrkB promotes ovarian cancer cell migration and invasion and it is decreased in TrkB knockdown cells while enhanced apoptosis.^202^ BDNF/TrkB inhibits the anoikis in human ovarian cancer cells via PI3K/AKT, generating chemoresistant cells.^206^

The body distribution of blood vessels and nerve fibers is similar, usually in a parallel manner. They share guiding molecules and signaling mechanisms that promote the growth of axons and blood vessels.^207^ This suggests that during cancer, the molecules that promote angiogenesis could also induce axonogenesis/neurogenesis mechanisms and vice versa. In fact, tumors are innervated mainly by sympathetic fibers,^208^ this correlates with the high parallelism of sympathetic nerves and body vasculature.

In gynecological cancers such as ovarian, cervical, uterine, fallopian tubes, vulvar, vaginal, and gestational trophoblastic neoplasms, neurotrophins strongly promote tumor-angiogenesis.^205^ NGF and BDNF can promote angiogenesis independently of VEGF (showed in Fig. 3); these factors could explain the resistance of tumors to anti-VEGF therapy. In preclinical models, BDNF promotes tumor growth by neovascularization, in a murine tumor model produces large and hyper-vascularized tumors (BNL cells in nude mice). BDNF overexpression in endothelial cells increases proliferation and vascularization (sensitive to the TrkB inhibitor K252a), while in patient samples with hepatocellular carcinoma (HCC), BDNF and TrkB are overexpressed, in fact, high expression of TrkB correlates with low patient survival.^209^ BDNF has high angiogenic potential by recruiting bone-marrow-derived cells as endothelial progenitor cells and pro-angiogenic hematopoietic cells (Sca-1^+^CD11b^+^)^210^ and induces differentiation of stem cells to endothelial cells.^211^ These effects may be relevant in tumor angiogenesis and vasculogenesis.

Antagonism or inhibition of Trks could potentially prevent the communication induced by neurotrophins, avoiding relevant processes in cancer: neuroplasticity involved in neuropathic pain, cancer cell proliferation, tumor-axonogenesis, and tumor-angiogenesis. In 2018, the FDA approved an inhibitor of Trk receptors, larotrectinib (Vitrakvi) for therapy of tumors with NTRK gene fusions.^212,213^ While in 2019 was approved entrectinib (Rozlytrek), a potent ATP-competitive inhibitor for Trks.^205,214^ It is necessary to continue the research on the role of neurotrophins in cancer and the effects of Trk inhibitors in conjunction with conventional therapy.

### Somato-sensorial nervous system: afferent fibers contribution to cancer progression

Sensorial afferent neurons not only sense proprioception and pain, but also modulate vascular and immune systems. In cancers associated to the nervous system, tumor cells grow taking advantage of sensory fibers which also enable cancer cells to invade the peripheral and central nervous systems. Spinal neuroinflammation detected by GFAP accompanies early stages of pancreatic ductal adenocarcinoma. In these conditions, nervous system damage is likely triggered by tumor-derived factors, and then tumor cells invade sensory neurons and migrate towards DRG and the spinal cord. Ablation of sensory neurons (C fibers mainly) in a neonatal mouse model (induced by capsaicin) prevents PNI and improves survival.^215^ Neuroinflammation triggered by tumor-associated macrophages also contributes to neuropathic pain.^216^

In tumors, there is communication among neurons, endothelial and cancer cells. Afferent fibers-derived peptides as substance P, commonly associated with inflammatory pain, are released on demand after secretion of primary afferent terminals adjacent to peripheral blood vessels by the antidromic depolarization. Substance P activates Gq- and Gs-coupled receptors such as NK1, promoting local endothelium-dependent vasodilation. In cancer, this process propitiates tumor progression by enhancing the availability of oxygen and nutrients. In addition, afferent nervous terminals and mast cells induce local vasodilation and inflammation through SP/calcitonin gene-related peptide (CGRP) and histamine, respectively. Communication networks among afferent fibers, mast cells and vessels exacerbate the tumor microenvironment. Mast cells are activated by SP to release vasodilators such as histamine that activate GPCRs in vascular smooth muscle cells to promote vasodilation.^217^ Furthermore, in murine melanoma models in which mast cells are sensitized with IgE, this atopic status contributes to tumor growth. In mast cells, the signaling pathways activated by the IgE/FcεRI/Fyn complex induce VEGF synthesis and secretion, contributing to tumor-induced angiogenesis^218^ (Fig. 2). GPCRs such as CB2 and GPR55 activated by anandamide and specific agonists inhibit mast cell degranulation.^219^

Tumors are innervated by sensorial fibers, in papillary thyroid cancer are detected peptidergic (sensorial fibers) and cholinergic (parasympathetic fibers) innervations, although most nerves are commonly adrenergic (sympathetic fibers).^208^ Direct communication between sensorial afferent fibers and cancer cells stimulates proliferation and invasion of transformed cells. In breast cancer cells, NK1 receptors, stimulated by SP, activate secretory pathways that increase extracellular activity of metalloproteinases (MMPs); turning on HER2 growth factor receptor transactivation which, via Src, elicits proliferative and invasive processes.^220,221^ In fact, cancer cells activate autocrine circuits by releasing SP, and in a preclinical tumor model with breast cancer cells (MDA-MB-231 and MDA-MB-453), NK1 antagonist (L-733,060) inhibits the tumor growth and synergizes with anti-HER2 therapies (AG825, AG1478 or lapatinib inhibitors)^176^ (Fig. 3). These findings suggest that NK1 antagonists could prevent growth factor receptor transactivation, restricting proliferation, but also preventing the effect of SP on mast cells and tumor peripheral vessels.

Hematopoietic growth factor receptors G-CSFR and GM-CSFRα are expressed in sensorial nerves. Bone metastasis from pancreatic carcinoma release granulocyte- and granulocyte-macrophage colony-stimulating factors (G-CSF and GM-CSF), promoting cancer pain. In sensory nerves, JAK/STAT3 signaling induces CGRP release and sprouting nerves and hypertrophy. Antibodies against G-CSFR or GM-CSFRα and JAK inhibitor (AG490) reduce tumor size, neurite outgrowth, and cancer pain.^222^

Emerging research highlights the contribution of cancer-derived exosomes to induce tumor axonogenesis, particularly by innervation of sensorial fibers.^186,223^ Head and neck squamous cell carcinomas (HNSCCs) are innervated by sensory nerves (TRPV1^+^, Transient Receptor Potential Vanilloid-type 1), but not by sympathetic (TH^+^, Tyrosine Hydroxylase) or parasympathetic nerves (VIP^+^, Vasoactive Intestinal Polypeptide).^186^ Tumor released exosomes containing EphrinB1 (EphB1, an axonal guidance molecule) induce sensory innervation of the tumor (Fig. 3). EphB1 is a transmembrane protein that activates the Eph receptor tyrosine kinases. Indeed, EphB1 knock out or the truncated extracellular domain partially prevents exosome-dependent axonogenesis.^186^

In murine models of human papillomavirus-induced head and neck cancer, tumor innervation is attenuated by inhibiting exosome release using Rab27A/B-deficient mEERL cells (Rab27A^−/+^ Rab27B^−/−^) or pharmacological blockade of mature exosome release by neutral sphingomyelinase inhibitor (GW4869).^186^ In addition, exosomes from colorectal cancer (CT26), melanoma (B16), and breast cancer (4T1) cells induce neurite outgrowth (PC12, rat pheochromocytoma cell line).^186^ Tumor-derived exosomes promote sensory innervation observed in human cervical cancer (TRPV1^+^ sensory nerves). Similarly, cervical cancer cell lines (Caski, HeLa, SiHa, and C66-3) release exosomes promoting neurite outgrowth (neuritogenesis).^224^

In oral cavity squamous cell carcinoma (OCSCC) high neural density has been detected and correlates with poor survival. p53-deficient cancer cells increase the tumor innervation by sensory nerves.^225^ Loss of p53, either by knock out or p53 mutants (p53^R273H^, p53^C238F^, and p53^G245D^) promotes the release of pro-axonogenic extracellular vesicles increasing sensory nerves infiltration. Conditioned media derived from human OCSCC cell line (HN31 cells with p53^C176F^ and p53^A161S^ mutations) contain EV and promote DRG neuritogenesis in vitro, while knock out of GTPases Rab27A and 27B prevented the effect.^225^

EV-derived axonogenic signals are triggered by loss of miR-34a. miRNA array of EVs derived from p53^WT^ cells and p53^null^ cells, revealed the loss of miR-34a and miR-141. In fact, miR-34a knock down or antagomiR-34a is enough to transform p53^WT^ cell-derived to p53^KO^ cell-derived EVs, promoting neuritogenesis.^225^ Moreover, p53-deficient head and neck tumors are enriched with adrenergic fibers and surgical lingual (sensorial) denervation decreased tumor volume and intratumor adrenergic fibers. miR-34a-deficient EVs regulates transdifferentiation of tumor sensory nerves to adrenergic (sympathetic) nerves that promote tumor growth.^225^ In human DRG or mouse TG sensory neurons, these EVs induce biosynthesis and release of noradrenaline. EVs increase sympathetic-associated genes expression and decreased sensory neuron genes.^225^

Altogether, the data indicate that miR-34a-less EV drives a sensory axonogenic switch and sensory nerve reprogramming to the adrenergic nerve.^225^ In the TME, the joint effect of soluble neurotrophic factors and pro-axonogenic EVs can lead to pro-tumor innervation during cancer progression, establishing a new pharmacologically modulable paradigm.^223^

### Autonomous nervous system (NA and ACh): efferent fibers contribution to cancer progression

In cancer patients, peripheral nerves that modulate autonomic responses promote tumor growth. This effect is mediated by infiltration of parasympathetic and sympathetic fibers within the tumor stroma. Acetylcholine and noradrenaline secreted by nervous terminals within the TME are recognized by their respective receptors in cancer cells, stimulating tumor progression.^190^ Prostate cancer studies have helped establish the participation of infiltrating autonomic fibers in cancer progression. Sympathetic nerve activity in the tumor is involved in early phases of the genesis of neoplasia^190^ and for the angiogenic switch;^226^ while in later phases the parasympathetic nerves promote invasion and metastasis (Fig. 3 insert).^6,190^ Another putative mechanism is linked to the immunomodulatory role of autonomic mediators which decrease anti-tumor immune responses. Neuropathic pain suffered by cancer patients is exacerbated by the aberrant communication between autonomic and sensorial fibers; antidromic spontaneous shots of sensorial fibers can enrich the TME. Autonomic fibers have a role in allodynia during neuropathic pain, since they are able to abnormally innervate to sensorial fibers, which propitiates their spontaneous activation triggering pain^227^ (Fig. 4).

### Sympathetic nervous system: noradrenaline and adrenaline as pro-tumor mediators

Chronic stress promotes cancer growth. Noradrenaline, the main neurotransmitter released by sympathetic fibers, plays a relevant role in stress responses. Stress seems to be particularly relevant in pancreatic cancer since pancreas is densely innervated by sympathetic fibers.^160,228^ In pancreatic cancer orthotopic and non-orthotopic murine models, bigger tumors are developed in animals subjected to continuous stress. Moreover, anti-stress pharmacological treatment attenuated pancreatic cancer progression.^228^ Pancreatic tumor growth is directly stimulated by isoproterenol, a β-adrenergic receptor agonist, likely stimulating β1 and β2 adrenergic receptors, both expressed in pancreatic cancer cells. Consistent with the pro-oncogenic role of these Gs-coupled receptors, constitutively-active Gα_s_ mutant exacerbates Ras-dependent pancreatic cancer.^52^ In addition, stress increases MMP-2 and MMP-9 expression in tumor and stromal cells to invade adjacent tissues. Very likely, direct β-adrenergic activation of pancreatic stromal cells, as stellate cells, affects tumor growth. Pancreatic stellate cells are like pancreas-specific fibroblasts that contribute to inflammation and carcinogenesis.^228^ Also, in a model of acute lymphoblastic leukemia, chronic stress increased cancer cell dissemination via β-adrenergic signaling (sensitive to propranolol). In this case, the effect seems to be mediated by the response of cells from the bone marrow.^229^

Sympathetic innervation on endocrine organs inevitably contributes to systemic effects of stress. Adrenal secretion of adrenaline plays an evident role in cancer. Adrenaline stimulates myeloma cell proliferation by activating β1- and β2-adrenoceptors, as indicated by the anti-proliferative effect of propranolol, a β-blocker.^230^ In a model of chemically induced hepatocarcinogenesis, adrenaline promotes cancer cell proliferation and survival triggered by β2-adrenergic signaling. It also inhibits autophagy and promotes HIF-1α stabilization stimulating gene expression of angiogenic factors. Adrenaline effect on tumor cells is inhibited by β2 antagonists (ICI-118,551 and butoxamine) and by receptor knockdown. Moreover, inhibition of β2-adrenergic signaling improved sorafenib effects, a small molecule inhibitor that targets VEGFRs, PDGFR, and RAF kinases.^231^

In OCSCC patients, p53-deficient tumors have high adrenergic nerve density (TH^+^) and correlate with low recurrence-free survival and lower overall survival rates. Sensory reprogramming to adrenergic in p53-deficient tumors in mice treated with a non-selective blocker of β1, β2, and α1 adrenergic receptors (carvedilol) inhibits growth and proliferation.^225^

Adrenergic signaling mediated by α1-adrenergic GPCRs also exhibits pro-tumorigenic properties. According to preclinical studies in cell cultures, pharmacological modulation (antagonists) of these receptors decreases proliferation, migration, and adhesiveness of prostate cancer cells. The α_1A_-AR subtype is expressed in androgen-sensitive prostate cancer cell lines (Rv1 and LNCaP cells); in contrast, α_1B_ and α_1D_ subtypes are only expressed in androgen-independent cancer cell lines (PC3 and DU145). Experimental evidence shows that α_1D_-adrenoceptor induces prostate cancer cell proliferation and migration.

In prostate cancer patients, high adrenergic nerve densities correlate with low recurrence-free survival,^190^ hence the communication between nerves and endothelial cells could be an interesting target for cancer therapies. Surprisingly, chemical (6-hydroxydopamine, 6-OHDA) and surgical (hypogastric nerve cut) sympathectomy inhibit the initiation of prostate tumors^190^ and progression of lung cancer in murine models (HCC827 and H446 cells).^160^ Interestingly, β2/3 receptors are relevant in tumor stromal cells, in mice lacking the β2 or β3 receptor, there is a delay in tumor growth, while double KO shows an exacerbate phenotype, arrest in tumor growth and angiogenesis.^190,226^ It has been characterized that intratumor adrenergic nerves induce the angiogenesis switch through endothelial stimulation by metabolic adjustments in prostate cancer. The sympathetic fiber-derived noradrenaline in the tumor activates β2-signaling in endothelial cells and inhibits the expression of the mitochondrial cytochrome c oxidase assembly factor COA6, consequently decreasing oxidative phosphorylation and activating the angiogenic switch. Therefore, inhibition of β2 adrenergic signaling in the tumor decreases vascularization and tumor growth.^226^ Indirectly, sympathetic fibers-derived noradrenaline promotes tumor neovascularization via VEGF expression and secretion from polarized M2-macrophages. This is prevented by chemical denervation and by the antagonist propranolol thus inhibiting lung tumor growth in mice.^160^

The integration of the available information suggests that axonogenesis and neurogenesis trigger angiogenesis induced by adrenergic signaling. The suggested sequential processes are pharmacologically adjustable. First, tumor-derived neurotrophins induce axonogenesis of adjacent autonomic fibers and/or the recruitment of neural progenitors, as described later in Section “Tumor neurogenesis: CNS-derived neural progenitor cells infiltrated in tumors”. Then, new intratumor sympathetic fibers activate the angiogenic switch induced by noradrenaline and neurotrophins on endothelial cells adjacent to tumor and pro-angiogenic macrophages.

### Parasympathetic nervous system: acetylcholine as a pro-tumorigenic mediator

Parasympathetic neurogenesis is strongly associated with tumor budding (presence of tumor cells isolated or in small groups located in the infiltrating front of the tumor) in patients with pancreatic ductal adenocarcinoma (PDAC). This process correlates with poor prognosis as it correlates with cancer aggressiveness and lower survival,^232,233^ particularly, high M3 receptor expression correlates with poor prognostic and tumor budding.^234^ In prostate cancer patients, high cholinergic nerve densities correlate with low recurrence-free survival.^190^ Vesicular acetylcholine transporter (VAChT) is the usual marker of parasympathetic neurogenesis and it is usually quantified by immunostaining,^235^ and its hypothetical pharmacological regulation could alter tumor growth.

Prostate tumors are infiltrated by parasympathetic cholinergic fibers that promote cancer dissemination; in contrast to the early contribution of sympathetic signaling, cholinergic signaling is relevant in late stages for invasion and metastasis. Pharmacological or genetic blockade of parasympathetic cholinergic signaling (M1, muscarinic receptor) decreases the metastasis of prostate cancer cells (Fig. 4).^190^ M1 receptor in tumor stroma promotes aggressiveness of prostate cancer, carbachol (a muscarinic agonist) enhances prostate cancer xenografts (PC-3) invasion of lymph nodes and is prevented by nonselective muscarinic (scopolamine) or M1 specific (pirenzepine) antagonists.^190^

Acetylcholine promotes proliferation and invasion of poorly differentiated non-small-cell lung carcinoma as demonstrated by the inhibitory effect of ionotropic acetylcholine receptor antagonists, particularly those that target heteropentameric α5 nAChR (α-conotoxin and mecamylamine) or α7 nAChR homopentameric (α-bungarotoxin) receptors.^236^ These nicotinic acetylcholine receptors (nAChR) belong to the neuronal group of ionotropic receptors activated by acetylcholine (5 α7 or, α2 α4 and 3 β2); which are structurally related to muscle nicotinic (2 subunits α1, β1, δ, and γ) receptors. All of them are ligand-dependent ion channels that allow Na^+^ entry, leading to cell depolarization. Expression of nicotinic receptors: α5, α7, β2, and β4 subunits, has been identified in lung carcinoma tissue samples.

### Tumor-derived neurogenesis: transdifferentiation of cancer stem cells

Neuron-like cells have been observed in peripheral tumors. They seem to be part of pathological mechanisms linked to the differentiation of cancer stem cells (Fig. 4). In the case of gastric and colorectal cancer, stem cells differentiate and acquire diverse phenotypes, mainly of autonomic neurons expressing VAChT (a marker of parasympathetic neurons), or TH (Tyrosine hydroxylase, characteristic of sympathetic neurons). They also express MAP2 (MAP2, Microtubule Associated Protein 2), which is restricted to cancer stem cells (CSC) with neural differentiation capacity within the tumor. By knocking down MAP2 it has been revealed that these undifferentiated cells generate functional autonomic neurons that stimulate tumor growth. The knocking down of MAP2 decreased the generation of neurons from human gastric and colorectal cancer stem cells and reduced the growth of tumor xenografts derived from human colorectal cancer stem cells.^235^ Similarly, as characterized in glioblastoma, tumor stem cells differentiate to endothelial-like cells forming vessels that irrigate tumors.^237,238^ Destroying cancer stem cells, as well as pharmacological inhibition of cancer stem cell differentiation, could prevent cancer progression. In order to design specific drugs suitable to inhibit aberrant cell differentiation into tumor-accelerating phenotypes, similar to neurons or endothelial cells, it is important to identify factors and conditions that lead cancer stem cell transdifferentiation, so they could be regulated with therapeutic goals in mind.

### Tumor neurogenesis: CNS-derived neural progenitor cells infiltrated in tumors

Tumor-induced neurogenesis occurs in prostate cancer through neural progenitor cell migration and differentiation of neurons into tumors (Fig. 4). The central nervous system (CNS)-derived neural progenitor cells (NPC) are recruited by prostate cancer cells to the primary tumor and metastasize in early stages, where initiate neurogenesis, generating adrenergic neurons mainly.^239^ This is consistent with the sympathetic contribution in early phases demonstrated in prostate cancer.^190^ Neural progenitor cells (marker doublecortin^+^, a microtubule-associated protein) from neurogenic regions of the brain (subventricular zone, SVZ) cross the blood–brain barrier and travel by the bloodstream until they infiltrate the tumors where they differentiate.^239^

The study of tumor neurogenesis in gastrointestinal malignancies is necessary, considering that enteric neural progenitor cells are more efficient in generating neurons (e.g. colon) than brain-derived progenitor cells.^240^ While in glioblastoma, brain tumor stem cells produce tumor neurogenesis, but also have tumor initiation capacity. The migration of both cells requires PI3K/AKT and Cdc42 activation and the inhibition of small GTPases, and PI3K prevents the migration and invasive capacity.^241^ CXCL12/CXCR4 signaling is relevant to NPC migration, and probably participates in tumor recruitment. In the opposite direction, glioblastoma stem cells invade SVZ through CXCL12/CXCR4; the tropism induced by CXCR4 activation can be avoided using antagonists (AMD3100 and PRX177561).^241^

Altogether axonogenesis and neurogenesis increase nerve density around the tumor and the number of dorsal root ganglion neurons in human prostate cancer^242^ and pancreatic cancer tissue compared to normal tissue.^188^ Prostate cancer cells overexpress semaphorin 4F (Sema4F) and its knock down inhibits the tumor axonogenesis and neurogenesis in vitro.^242^ Additionally, overexpression of Sema4F induces proliferation and migration of prostate cancer cells (DU145) and correlates with a recurrence-free survival of patients with prostate cancer.^243^ Sema4F may contribute to the communication between fibers and cancer cells for this to ultimately migrate along fibers.

Granulocyte colony-stimulating factor (G-CSF) has neurotropic functions; in prostate tumor mice (Hi-Myc) G-CSF increased nerve outgrowth, invasion, and metastases. G-CSF induces new cholinergic parasympathetic nerve fibers in the orthotopic tumor for metastasized. Interestingly, G-CSF administration rescued the development of orthotopic tumor xenografts previously sympathectomized with 6-OHDA, protected sympathetic neurons.^244^ G-CSF may promote the recruitment of neural progenitor cells to increase sympathetic cells in the tumor. The tumor-derived factors that particularly promote the migration of neural progenitor cells are unknown, and it is necessary to deepen into the characterization of tumor mediators that induce the migration and differentiation of NPCs to develop small molecules that inhibit the tumor neurogenesis process. Neurotrophins^17^ and extracellular vesicles^225^ enriched in the tumor possibly promote the differentiation of neural progenitor cells to tumor-associated sensory and autonomic fibers. Tumor axonogenesis and neurogenesis are potential targets for cancer therapy.

## Classical central nervous system neurotransmitters (dopamine, glutamate, and GABA) impact cancer progression

### Systemic dopamine in tumor progression

Dopamine (DA), a monoamine neurotransmitter characteristic of the CNS, affects the interplay between the immune and central nervous systems. Therefore, immune responses controlled by DA mediate the effects of this neurotransmitter in cancer progression. Immune cells, including B lymphocytes, NK cells, monocytes, macrophages, dendritic cells, neutrophils, effector and regulatory T cells express dopamine receptors,^245^ which are either coupled to Gs (D1R and D5R) or Gi (D2R, D3R, and D4R). Dopamine is produced not only by the brain, but also by peripheral organs including the digestive tract, spleen, and pancreas.^246^ Peripheral dopamine modulates anticancer immune responses. In patients with lung cancer, dopamine plasma levels increase up to 5-fold, reaching immunomodulatory concentrations that, in vitro, inhibit T cell proliferation and cytotoxic capacity. This effect, demonstrated in samples from normal donors and cancer patients, occurs via D1 receptors, sensitive to SCH23390 (D1R/D5R antagonist).^247^

Some cancer cells are directly affected by DA. For instance, dopamine inhibits osteosarcoma cell proliferation via downregulation of ERK1/2 and PI3K/AKT pathways, controlled by D1 receptors.^248^ In the case of gastric cancer cells, DA inhibits migration and invasion, potentially via inhibition of the EGFR-AKT pathway.^249^ In gastric cancer, increased expression of D2R negatively correlates with patient survival.^250^ Similarly, increased levels of D2R have been detected in samples of cervical,^251^ lung,^252^ and breast cancer.^253^ In preclinical cancer models, dopamine receptors are being studied as potential drug targets. In murine lung cancer, D2R agonist inhibits angiogenesis, limiting tumor advance, although the mechanism remains to be clarified.^252^ This suggests that D2R agonists may be useful adjuvants in anti-tumor conventional therapy to lung carcinoma.^254^ Paradoxically, D2R antagonists as trifluoperazine and thioridazine eradicate cancer stem cells (CSC) in breast and lung cancer, cells that resist and survive conventional therapy.^255^ Given the contribution of cancer stem cells (CD133^+^) to carcinoma progression, the finding that they overexpress D2R raises interesting opportunities for pharmacological intervention. Control of this system in cancer stem cells reduces proliferation and invasiveness by suppression of ERK and AKT pathways and down-regulation of MMP9 and octamer-binding transcription factor 4 (Oct-4).^254^ These effects on cancer stem cells support the possible use of antipsychotic drugs as co-adjuvants to existing therapy.^255^ Although further evidence is required to assure the role of dopamine receptors against CSC survival, this offers a new promise for cancer treatment. In addition, other dopamine receptors might be relevant targets. Accumulating evidence includes D4R, which has also been found overexpressed in breast cancer, and D5R which, according to preclinical studies, promotes autophagy leading to cell death via increased ROS production and inhibition of the mTOR pathway.^256^

### Glutamate metabotropic and ionotropic receptors in cancer

**Fig. 5:**
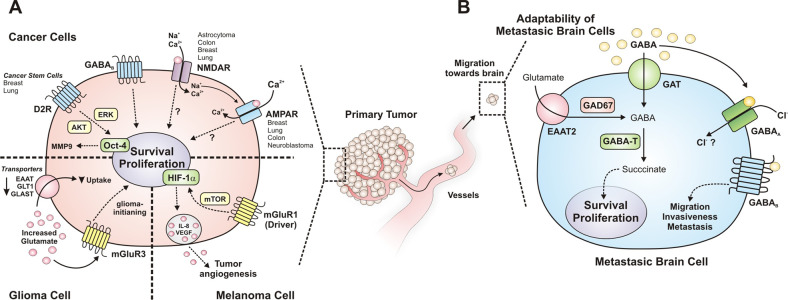
Glutamate, GABA, and dopamine contribution to cancer progression. **a** Glutamate stimulates tumor cell proliferation and survival via metabotropic (mGluR3, mGluR1) and ionotropic (NMDAR and AMPAR) receptors; particularly, mGluR1 overexpression drives melanoma. Direct effects of dopamine and GABA on cancer cells promote survival and proliferation via D1/5 and GABA_B_ receptors, respectively. Glutamate release is increased in glioma cells. Dopamine induces survival and proliferation of cancer stem cells (CSC) via D2 receptor, anti-psychotic drugs decrease the CSC population. **b** Metastatic brain cells from breast cancer acquire GABAergic properties to take advantage of GABA and glutamate neurotransmitters sustaining their metabolism and survival. GABA and glutamate are uptaken by GAT and EAAT2, respectively, in metastatic cells; the neurotransmitters are metabolized. In addition, GABA receptor activation in cancer cells promotes metastasis. Blockers for GABA (GAT) and glutamate (EAAT2) transporters and GABA antagonist could inhibit the survival and proliferation of metastatic cancer cells

Glutamate (Glu), a paradigmatic excitatory neurotransmitter in the mammalian brain, is tightly controlled to prevent neuronal death due to excitotoxicity. Altered glutamatergic signaling has been linked to several neurodegenerative diseases and disorders, as well as oncogenic and metastatic processes in glioma (the most common type of primary brain tumors), such as glioblastoma, the most frequent and lethal cancer of the central nervous system;^257^ among other cancer types.^258^ Increased glutamate levels occur in glioma and astrocytomas (Fig. 5a). In vitro, malignant cells release enough glutamate to reach neurotoxic concentrations. Moreover, aberrant glutamatergic signaling in glioma has also been linked to decreased glutamate uptake due to poor glutamate transporter activity (EAAT, excitatory aminoacid transporter), or expression (GLT1, glutamate transporter-1 and GLAST, glutamate/aspartate transporter).^258,259^

Although high levels of extracellular glutamate are toxic for normal neurons; this neurotransmitter has neurotrophic effects in GBM.^257^ Glutamate is the natural agonist of ionotropic (iGluRs, which are ion channels) and metabotropic (mGluRs, G protein-coupled) receptors. The first group includes: *N*-methyl-d-aspartate receptors (NMDAR), a-amino-3-hydroxy-5-methyl-4-isoxazolepropionic acid receptors (AMPAR), and kainate receptors (KAR); the second is composed by eight G protein-coupled receptors (mGluR1, R2, R3, and R5 are Gq protein-coupled; while mGluR4, R6, R7, and R8 are coupled to heterotrimeric Gi proteins).^260^ In chemotherapy-resistant glioma cells, mGluR is considered an oncogene. In vitro, a mGlu3R agonist (LY379268, mGluR2/3 agonist) maintains glioma-initiating cells in the undifferentiated state, whereas a mGlu3R antagonist (LY341495) induces differentiation to astrocytes. In vivo mGlu3R antagonists limited brain tumor growth or infiltration in nude mice models.^261^

In non-neuronal cancer cells, genomic and proteomic studies have revealed glutamate receptor mutations and aberrant glutamatergic signaling related to iGluRs, mGluRs, and their downstream effectors.^258,262^ Known to be absent in normal melanocytes,^263,264^ mGluR1 was detected in 7 out of 19 biopsies from melanoma patients, and in 12 out of 18 melanoma cell lines.^264^ These findings suggested a potential role for mGluR1 in melanoma progression. In mouse models, stable melanocyte expression of mGluR1 is sufficient to transform cells, enabling aggressive tumorigenic properties.^263–265^ Overexpression of mGluR1 generates larger and aggressive murine melanoma tumors with increased blood vessels. Mechanistically, mGluR1-dependent activation of the PI3K/AKT/mTOR/HIF1 pathway increased the content of IL-8 and VEGF in the tumor microenvironment, promoting proliferation, survival and angiogenesis^266^ (Fig. 5a). In this model, an inhibitor of glutamate release (riluzole), prevented tumor growth. Riluzole is being assessed, with promising results, in advanced melanoma patients included in phase II clinical trials.^267^ Reminiscent of uveal melanoma driven by mutant Gq/11,^51^ which in preclinical studies is sensitive to FR900359,^268^ the mGluR1/Gq signaling pathway emerges as a potential target in melanoma.

NMDAR has been detected in different tumors.^269^ Specifically, the NR2B subunit is increased in biopsies from glioma, pancreatic ductal carcinoma, breast and ovarian cancer.^270^ Accordingly, different human cancer cell lines exhibit NMDAR functionality. A paradoxical role of NMDAR in cancer has been revealed by the anti-tumor effects of both agonists as well as antagonists. These intriguing observations are likely linked to different signaling pathways being effective in different cancer types.^271^ In fact, NMDAR antagonist (MK-801 or dizocilpine) and AMPA antagonist (GYKI52466) inhibit cancer cell proliferation and invasion, whereas they are innocuous in human skin fibroblasts and bone marrow stromal cells^271,272^ Dizocilpine was effective against colon adenocarcinoma (HT29), astrocytoma (MOGGCCM), breast carcinoma (T47D), and lung carcinoma (A549), whereas AMPA antagonist (GYKI52466) was effective in breast carcinoma (T47D), lung carcinoma (A549), colon adenocarcinoma (HT29), and neuroblastoma (SKNAS). Indicative of their potential in combined therapies, glutamate antagonists improve the effect of cytostatic drugs.^272^

NMDAR behaves as a tumor suppressor as its agonists inhibit cancer cell proliferation by interfering with mTOR and ERK signaling pathways.^271^ Accordingly, multiple cancer cell lines including gastric cancer lines,^273^ human esophageal cancer^274^ and non-small-cell lung cancer cell lines^275^ show decreased expression of NMDAR2B subunit. In fact, epigenetic control of the NR2B subunit promoter, by methylation, is being postulated as a biomarker in gastric cancer. In these cancers, NMDA suppresses disease progression. NMDAR activation triggers the internalization of CAT 1 and 3 (cationic amino acid transporters). Therefore, in response to low levels of intracellular arginine, AMPK inhibits mTORC1.^276^ Also, NMDA-dependent increase of cytosolic calcium activates calcineurin which in turn activates STEP (striatal enriched protein tyrosine phosphatase), inhibiting ERK1/2^277^ as part of the anti-mitogenic mechanism. Given the antiproliferative effects of NMDAR agonists, clinical trials and precise patient selection likely will validate the therapeutic potential of these emerging antineoplastic agents.

### GABAergic system modulates breast cancer metastasis and prostate cancer

GABA (γ-aminobutyric acid), an inhibitory neurotransmitter that activates agonist-gated ionotropic channels (GABA_A_-R, allowing Cl^−^ efflux), and Gi-protein coupled receptors (GABA_B_-R), is widely distributed in the CNS and other peripheral tissues. Given the high prevalence of cancer metastasis to the brain, GABA (as other neurotransmitters), has been studied as a potential oncometabolite helping to establish metastatic niches in the brain.^278^

In the case of breast cancer patients, the brain is commonly colonized by tumor cells. Interestingly, perhaps as an adaptability mechanism, metastatic breast cancer cells invading the brain express GABA_A_-R and a repertoire of related proteins similar to those of GABAergic neurons, including GABA transaminase (GABAT), glutamate decarboxylase (GAD67), GABA transporter, reelin, and parvalbumin.^279^ The 15 isoforms of the GABA receptor mRNA, as well as vesicular GABA transporter (VGAT), GABA 1 to 3 transporter (GAT1-3) and the betaine-GABA transporter (BGT) expression are highly regulated in HER2^+^ breast cancer brain metastases. Therefore, metastatic tumor cells acquire GABAergic machinery enabling them to survive and proliferate in response to GABA. These acquired abilities are independent of GABA_A_ receptor signaling, as indicated by the proliferative effect of GABA even in the presence of muscimol, a GABA_A_ antagonist. GABA-induced proliferation of metastatic tumor cells is attributed to their gained ability to uptake and catabolizes GABA, producing succinate and NADH as a biosynthetic source (Fig. 5b). Consistent with this possibility, the proliferative effect of GABA on metastatic cells is abolished by vigabatrin (GABA transaminase inhibitor). In addition, some metastatic tumor cells in the brain overexpress GAD67, an enzyme that converts glutamate to GABA, sustaining an additional metabolic source to promote cancer proliferation in the brain.^279^ Therefore, GABA transaminase and other proteins linked to the acquired ability of metastatic cells in the brain to feed on GABA and glutamate, as sources of biosynthetic energy, emerge as potential therapeutic targets to treat metastatic breast cancer.

Out of the central nervous system, GABA plays a role in cancer. For instance, GABA_B_ receptors enhance the aggressive behavior of metastatic breast cancer cells invading the lungs. A mice model of breast cancer showed that a GABA_B_-R agonist potentiates lung metastasis without affecting primary tumor volume, whereas an antagonist decreased metastases.^280^ Baclofen, a GABA_B_-R agonist, induces migration, invasion, and metastasis mediated by ERK1/2; in contrast, a GABA_B_-R antagonist (CGP55845) decreased migration and invasion. GABA is a neurotrophic factor effective during neural crest development and exhibits similar effects in neural crest-derived chondrosarcoma. In a human chondrosarcoma cell line, GABA promotes proliferation; in contrast, a GABA_B_-R antagonist (CGP54626) induced apoptosis by inhibition of the PI3K/AKT/mTOR and MAPK pathways and activation of caspases 3 and 9.^281^ These results encourage the characterization of GABA_B_ drugs as potential co-adjuvants of current chondrosarcoma therapy.

In prostate cancer^282,283^ GABA_A_-R is expressed in cancerous gland tissue, but absent normal tissue. Thus, this chlorine channel brings about the GABAergic system to aggravate the situation in prostate cancer. Consistent with this role, GABA and isoguvacine (GABA_A_-R agonist) increased prostate cancer (PCa) cell proliferation, particularly in the case of two cell lines: PC-3 cells (bone metastasis, androgen-independent) and LNCaP (lymph node metastasis androgen-dependent). The proliferative mechanism activated by GABA_A_-R in prostate cancer cell lines is likely mediated by transactivation of EGFR and Src-dependent proliferation.^282^ In fact, 3α-diol neurosteroid, synthetized from 5α-dihydrotestosterone by AKR1C3 (Aldo-keto reductase family 1 member C3), induces PC-3 cell proliferation and promoted growth of large vascularized tumors in a GABA_A_-R-dependent manner. 3α-diol is an allosteric modulator of GABA_A_-R, increasing EGF expression and subsequent activation of EGFR, leading to an increase in cell proliferation. These effects are prevented by GABA_A_-R antagonists (dihydroergotoxine mesylate, picrotoxin, or bicuculline methobromide picrotoxin). Therefore, inhibition of AKR1C3 and GABA_A_-R in prostate cancer would hypothetically potentiate conventional therapy.^283^

## Conclusions

**Fig. 6:**
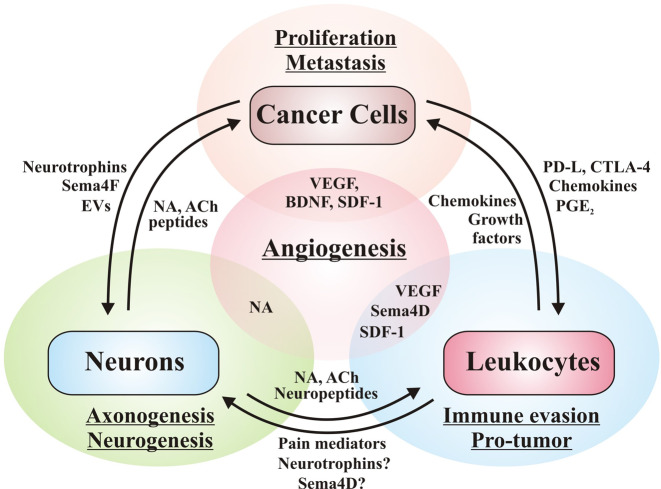
Positive regulation among cancer cells, leukocytes, and neurons to cancer progression. Cancer cells release neurotrophins and extracellular vesicles to activate the neurogenic/axonogenic switch and release chemotactic factors while expressing ligands in the membrane to recruit and activate the immunosuppressor switch in the tumor microenvironment for reciprocally stimulate the cancer cells and the angiogenic process. Cancer-associated nerves release peptides (sensorial fibers) and neurotransmitter as NA or ACh (autonomic fibers) stimulating proliferation and migration of cancer cells, but they also recruit and activate immunosuppressor leukocytes as M2-macrophages and MDSCs. Leukocytes release pain mediators and stimulate to nerves, but also release neurotrophins, inducing tumor innervation. Protumor factors are released by leukocyte populations promoting cancer cell proliferation, migration, and metastasis. Joint of cells activate the angiogenic switch, via angiogenic growth factors released by cancer cells and leukocytes as mast cells or M2 macrophages, and by sympathetic-derived noradrenaline. NA noradrenaline, ACh acetylcholine, VEGF vascular endothelial growth factor, BDNF brain-derived neurotrophic factor, SDF-1 Sema4D: semaphorin 4D, Sema4F semaphorin 4F, PGE_2_ prostaglandin E_2_. EV extracellular vesicles

Conventional antineoplastic therapies lack specificity and their high toxicity limits their efficacy. Ideally, they should be combined with more precise therapeutic molecules obtained by knowledge-based design. Therefore, current efforts aimed to achieve a deep understanding of the mechanistic basis of cancer pathophysiology are revealing novel therapeutic targets (Table 1). Cell communication is the focus of innovative therapies. Angiogenesis inhibitors have proven the concept; although they are effective in a limited number of cancer patients and resistance is an emerging problem. To face this challenge, it is important to continue evaluating cell communication in cancer, keeping in mind the complexity of the tumor microenvironment and the contribution of multiple cells and systems. In this review, some oncogenic communication networks among cancer cells, leukocytes, and neurons were discussed (Fig. 6), pointing out emerging targets, particularly receptors within the neuro-immune system (visualized from an integral perspective). These knowledge-based targets are essential players in the communication among different cells and systems, known to sustain cancer progression. The goal is to target them as co-adjuvants in cancer therapy to counteract those mechanisms by which cancer cells evade the immune system and those that promote axonogenesis, neurogenesis and PNI. New anti-neurogenic drugs and immunotherapies are an opportunity against cancer. In conclusion, as a paramount player in cancer progression, the neuro-immune axis is an important source of communication molecules and their receptors are being characterized as therapeutic targets that will revolutionize the efficacy and potency of conventional chemotherapies.

**Table 1:** Endogenous molecules promoting tumor growth and potential co-adjuvant therapeutic targets as disruptors of oncogenic cell–cell communication

Molecule and pro-tumor functions	Type cancer (line/cell target)	Genetic or pharmacological evidence, targets
**Cancer cell stimulation**		
**Noradrenaline/Adrenaline** α_1D_-adrenergic induced proliferation and migration^284^	Prostate cancer cells (PC-3).	α_1D_-adrenoceptor antagonist (A175).
β-adrenergic signaling induced by chronic stress in cancer cells, β2-adrenergic signaling promotes proliferation and survival^230,231^	Pancreatic ductal adenocarcinoma, acute lymphoblastic leukemia, hepatocarcinoma.	β1 antagonist (Propranolol), β2 antagonist (ICI118,551, butoxamine and propranolol) improved sorafenib effect.
β2/3-adrenergic signaling promote angiogenic switch by decreasing oxidative phosphorylation^190,226^	Prostate cancer (PC-3).	Chemical (6-OHDA) and surgical (hypogastric nerve cut) sympathectomy; β2/3-adrenergic knock out.
**Acetylcholine (ACh)** Cholinergic fibers promote prostate cancer invasion in late phases to metastasize via M1^190^	Prostate cancer cells (PC-3), ↑ stromal tumor.	Muscarinic antagonist (scopolamine), M1 antagonist (pirenzepine). M1 receptor KO.
Parasympathetic neurogenesis is strongly associated with tumor budding,^233^ particularly, M3 receptor overexpressed^234^	Pancreatic ductal adenocarcinoma (PDAC).	Unproven drugs for tumor budding.
Ionotropic acetylcholine receptor induces proliferation and invasion^236^	Non-small-cell lung carcinoma.	α5 nAChR antagonist (α-conotoxin and mecamylamine), α7 nAChR antagonist (α-bungarotoxin).
**Substance P (SP)** NK1 receptor induces transactivation of EGFR and HER in cancer cells^220^	Pancreatic cancer, breast cancer (MDA-MB-231 and MDA-MB-453), ↑vessels, ↑mast cells.	NK1 antagonist (L-733,060) synergizes with HER2 inhibitors (AG825, AG1478, or lapatinib).
**Glutamate (Glu)** mGluR1 overexpression drive melanoma through PI3K/AKT/mTOR/HIF1 pathway^265–267^	Cutaneous melanoma.	mGluR1 antagonist, inhibitor of glutamate release (riluzole).
mGluR3 maintains glioma-initiating cells in an undifferentiated state^261^	↑Chemotherapy-resistant glioma cells.	mGlu3R antagonist (LY341495).
NMDAR induce cancer cell proliferation and invasion^271,272^	Colon (HT29), astrocytoma (MOGGCCM), breast (T47D), and lung (A549).	NMDAR antagonist (MK-801 or dizocilpine).
AMPAR induce cancer cell proliferation and invasion^272^	Breast (T47D), lung (A549), colon (HT29), and neuroblastoma (SKNAS).	AMPAR antagonist (GYKI52466).
**γ-aminobutyric acid (GABA)** Tumor cell proliferation by metabolizing GABA and glutamate^279^	Breast cancer cell-brain metastasis (4T1).	GABAT inhibitor (Vigabatrin), transporter inhibitors, GAD67 inhibitors.
GABA_B_-R induces invasion and metastasis mediated by ERK1/2^280^	Metastatic breast cancer.	GABA_B_-R antagonist (CGP55845).
**Dopamine (DA)** D2R induces cancer stem cells (CSC) survival^254^	Breast and lung cancer (↑CSC)	D2R antagonist (trifluoperazine and thioridazine).
**Neurotrophins (NGF, BDNF)** In cancer cells, neurotrophins/Trk promotes survival, proliferation, migration, and invasion^202,205,212–214^	↑Ovarian cancer cells (OVCAR-3, SKOV-3, OVCA420 / 429 / 433).	TrkB knockdown, pan-Trk inhibitor (PLX-7486).
	Tumors with NTRK gene fusions.	Trk inhibitor (larotrectinib/Vitrakvi, entrectinib/Rozlytrek).
Neurotrophins strongly promote tumor-angiogenesis^205^ BDNF recruits to EPC and pro-angiogenic hematopoietic cells^210^	Gynecological cancers (↑endothelial cells). ↑EPC, ↑ Sca-1^+^CD11b^+^ cells.	TrkB inhibitor (K252a).
**Immune evasion and pro-tumor BMDCs**		
**PD-L1/PD-L2** Overexpression of PD-L1 in cancer, decrease of IL-2 and IFN-γ production, reduced T cell proliferation and cytotoxic effects^83,87^ Suppression of T-cell receptor (TCR) by SHP2 activation^81^	Melanoma, sarcoma, ovarian cancer T-cell non-Hodgkin lymphoma, hepatocellular carcinoma.	Anti-PD-1 (pembrolizumab). Anti-PD-1 antibodies in cases of drug resistance to cytotoxic chemotherapy.^87^ Anti-PD-1 synergize with a vaccine against hepatocellular carcinoma (GPC3-derived peptide)^96^
**B7 (CD80)** Activates to CTLA-4 in cytotoxic T lymphocyte and NK to suppress.^108^ Increase CD4^+^/FOXP3^+^ regulatory T cells^100,123^	Melanoma, head and neck sarcoma, colorectal cancer, lung cancer, renal carcinoma, mesothelioma (↓T cells, ↑Tregs).	Anti-CTLA-4 monoclonal antibodies (ipilimumab and tremelimumab).
**Prostaglandin E**_2_**(PGE**_**2**_**)** EP receptors induce differentiation and recruitment of MDSC^22^	Breast cancer (↑MDSCs)	COX2 inhibitor (SC58236), EP1/2 antagonist (AH6809), EP4 antagonist (AH23848).
**IL-1β** Promotes the accumulation of MDSC in tumor^285^	Breast cancer (↑MDSCs).	Physiological antagonist IL-1Ra, pathway inhibitors.
**CCL2** Induce colonization of nerves by endoneurial macrophages, relevant cells to perineural invasion^195^	Prostate and pancreatic cancer (↑ endoneurial macrophages).	CCR2 KO or KD. blocking anti-CCL2 antibody.
**TGFβ** Decreases innate and adaptive antitumor immune response^134^ Decrease NK cells^135^	Neuroblastoma and hepatocellular (↓NK).	TGFβ-RI inhibitor (Galunisertib (LY2157299), synergize with anti-GD2 (dinutuximab).
**CD25** Increase Tregs phenotype^133^	Metastatic melanoma (↑Tregs CD25 + ).	Anti-CD25 (Daclizumab) induces depletion of Treg cells.
**Dopamine (DA)** DA inhibits T cell proliferation and cytotoxic capacity via D1 receptors^247^	Lung cancer	D1/D5 antagonist (SCH23390).
**Semaphorin 4D (Sema4D)** Sema4D+ macrophages increase tumor growth by inducing angiogenesis and vessel maturation by binding to the plexin B1 receptor on endothelial cells^172^	Breast cancer murine model (TSA cells).	Anti-plexin B1 c-Met inhibitor (PHA-665752). Knock out sema4D
**Catecholamines (NA/AD)** M2 macrophages polarization and induce VEGF synthesis and secretion activating the angiogenic switch^160^	Lung cancer (HCC827 and H446 cells).	Chemical sympathectomy(6-OHDA); β-antagonist (propranolol).
**Tumor-derived factors** IL-10 and IL-4 cytokines of TME promotes polarization of M2 macrophages previously attracted by VEGF-A^158,159^	Breast cancer (↑M2 macrophages) VEGF-induced skin carcinogenesis (HaCaT) (↑M2 macrophages).	Anti-IL10R antibody IL-4Ra-blocking antibody
**Tumor axonogenesis/neurogenesis and perineural invasion**		
**Noradrenaline/Acetylcholine** Cancer cell migration along neurites^286^	Prostate cancer cells (PC-3).	β-antagonists (propranolol and penbutolol), M antagonists (atropine and hyoscine).
**Noradrenaline** β2-adrenergic induces perineural invasion via PKA/STAT3 signaling in cancer cells^192^	Pancreatic cancer cells (MIA PaCa-2 and BxPC-3).	β-antagonist (propranolol), PKA inhibitor (KT5720), STAT3 inhibitor (AG490).
β-adrenergic signaling induces NGF and BDNF to axonogenesis^287^	Pancreatic ductal adenocarcinoma.	β2 antagonists (ICI-118,551, propranolol).
**ACh** Parasympathetic signaling via M1 receptor in tumor stroma promotes invasion and metastasis^190^	Prostate cancer xenografts (PC-3).	M1 receptor knock out. Nonselective M antagonist (scopolamine), M1 antagonist (pirenzepine).
**TGFβ** Schwann cells releases TGF inducing aggressiveness and perineural invasion^200^	Pancreatic cancer (Capan-2).	TGFβ-RI inhibitor (SB-431542).
**CCL2** Nerve-released CCL2 induces cancer cell migration and PNI via CCR2 signaling^194^	Prostate cancer cell (PC-3, DU 145, and H292)	CCR2 KO or KD. Blocking anti-CCL2 antibody.
**CSF-1** Promotes recruitment of endoneurial macrophages to the tumor-promoting perineural invasion^195^	Melanoma, pancreatic cancer (↑M2 macrophages).	Anti-CSF-1R (emactuzumab in clinical trials). CSF-1 receptor blocker (GW2580).
**GDNF-GRFα1** Released by neurons, Schwann cells and endoneurial macrophages to promote perineural invasion^195,197^	Pancreatic adenocarcinoma.	GFRα1 co-receptor knock down. RET inhibitors (PYP1).
**Semaphorin 4F (Sema4F)** Overexpressed in cancer cells, promotes axonogenesis and potentially differentiation of NPC^242,243^	Prostate cancer (DU145), ↑autonomic fibers, ↑neural progenitor cells.	Sema4F knock down.
**G-CSF and GM-CSF** G-CSF increases nerve outgrowth, invasion, and metastases. Induces new parasympathetic and sympathetic fibers^244^	Prostate tumor (PC-3).	Unproven drugs.
In sensorial nerves, JAK/STAT3 signaling of receptors G-CSFR and GM-CSFRα, promotes cancer pain, CGRP release, and nerves’ sprouting^222^	Human pancreatic carcinoma Sarcoma (2472 fibrosarcoma cells)	anti-G-CSFR or anti-GM-CSFRα JAK inhibitor (AG490).
**Neurotrophins (NGF, BDNF)** Induce tumor axonogenesis of autonomic and sensorial fibers^287^	Pancreatic cancer (↑autonomic fibers, ↑NPC)	Pan-Trk inhibitor (PLX-7486).
**EphrinB1 exosomes** Cancer exosomes induce tumor innervation by sensorial fibers^186^ EV-derived axonogenic signals are triggered by loss of miR-34a^225^	Neck squamous cell carcinomas, colorectal cancer (CT26 cells), melanoma (B16 cells), breast cancer (4T1 cells) and cervical cancer (Caski, HeLa, SiHa, and C66-3).	Rab27A/B knock out Neutral sphingomyelinase inhibitor (GW4869 inhibits release of mature exosomes).
**Fiber-derived factors** Persistent pain (sensorial fibers) stimulate the tumor growth^46^	Breast cancer (Walker 256 carcinoma cells).	Anesthetic (bupivacaine), analgesic drug (morphine).
